# Biodegradable nanofibrous scaffolds enhance standard of care for glioblastoma via localized targeted therapy

**DOI:** 10.1016/j.jconrel.2025.114225

**Published:** 2025-09-11

**Authors:** Ryan N. Woodring, Elizabeth G. Graham-Gurysh, Sophie E. Mendell, Kevin E. Shilling, Nicole Rose Lukesh, Katie A. Hipp, William C. Zamboni, Eric M. Bachelder, Kristy M. Ainslie

**Affiliations:** a Division of Pharmacoengineering & Molecular Pharmaceutics, Eshelman School of Pharmacy, UNC, Chapel Hill, NC, USA; b Department of Biomedical Engineering, NC State/UNC, Chapel Hill, NC, USA; c Department of Microbiology and Immunology, School of Medicine, UNC, Chapel Hill, NC, USA; d Division of Pharmacotherapy and Experimental Therapeutics, Eshelman School of Pharmacy, UNC, Chapel Hill, NC, USA

**Keywords:** Glioblastoma, Synergy, Electrospinning, Polymer, Controlled release

## Abstract

Combination therapy is a well-established clinical strategy for treating aggressive cancers, but its success has not translated to patients with glioblastoma *multiforme* (GBM)—the most aggressive malignancy of the central nervous system. In this study, we evaluated the effects of combining temozolomide (TMZ), the standard chemotherapeutic agent for GBM, with several candidate targeted therapies to improve current outcomes in a mouse model of GBM resection and recurrence. In vitro, the EGFR inhibitor, erlotinib (ERL), emerged as the most promising combination drug across a diverse panel of GBM cells. In vivo, the therapeutic response was enhanced through localized delivery. ERL was encapsulated into electrospun acetalated dextran (Ace-DEX) scaffolds (Ace-ERL), a biodegradable and biocompatible polymer system that enables tunable degradation and controlled drug release. Local delivery of Ace-ERL to the resection cavity improved the pharmacokinetic profile and, when combined with systemic TMZ, significantly enhanced survival in a patient-derived xenograft mouse model. These findings support a novel translational approach to leverage combination therapy in GBM by pairing targeted delivery with standard chemotherapy.

## 1. Introduction

Glioblastoma *multiforme* (GBM) is a highly aggressive brain tumor that claims the lives of over 10,000 individuals in the United States each year. [1] Despite treatment with the current standard of care, including tumor resection, radiation, and chemotherapy, the recurrence rate remains nearly universal, with the five-year survival rate remaining stagnant below 7 %. [2] Although researchers have spent the last two decades investigating improved therapeutic approaches, no alternative has surpassed the median survival of 12 to 15 months in the clinic, underscoring the urgent need for new strategies that address the persistent shortcomings of the present approach. [3] As GBM incidence increases and the global market for treatment reaches $2.74 billion USD this year, innovative approaches are needed to overcome the historical limitations of newly developed therapies for GBM. [4]

A key limitation of the existing treatment is the widespread resistance to temozolomide (TMZ), the FDA-approved alkylating agent used as the standard of care for GBM chemotherapy, given orally for systemic delivery. TMZ induces cytotoxic double-stranded DNA breaks by methylating DNA at the O6-guanine position, leading to apoptosis. [5] However, the DNA repair enzyme O^6^-methylguanine-DNA methyltransferase (MGMT) can reverse this damage, reducing TMZ efficacy. When the MGMT promoter is hypermethylated, its expression is suppressed, making tumors more responsive to TMZ treatment. Unfortunately, less than half of GBM patients display MGMT promoter methylation, making them less responsive to TMZ. Additionally, excessive TMZ exposure may upregulate MGMT or other DNA repair pathways, further compounding resistance. [6]

Since TMZ’s approval in 2005, extensive molecular profiling efforts have advanced our understanding of GBM biology. A landmark initiative in 2008, The Cancer Genome Atlas (TCGA) project, sequenced over 200 GBM tumors and identified prominent mutations and pathway alterations in RTK/RAS/PI3K/AKT, RB1, and P53 signaling, highlighting promising new therapeutic targets. [7] Verhaak et al. (2010) built upon this assessment and identified four subtypes—Proneural, Neural, Classical, and Mesenchymal—with unique genetic signatures, thus paving the way for a precision medicine approach for GBM treatment. Further refinements to classification systems have incorporated glioma stem cell populations and tumor microenvironment features, expanding the number of clinical trials with novel targeted therapies. [8,9] However, GBM’s inherent intratumoral heterogeneity remains a major challenge, as a single tumor may harbor multiple subtypes or evolve. Consequently, monotherapies are unlikely to provide durable responses. [10]

Combination therapies that consider GBM heterogeneity as well as newly identified targets offer a promising way forward to treat GBM. By employing agents with distinct mechanisms of action, such regimens can disrupt multiple oncogenic pathways simultaneously while mitigating the development of resistance. [11] Although some clinical studies have investigated combinations of targeted therapies with TMZ, these efforts have yielded limited success—often due to poor study design, lack of biomarker-driven stratification, or insufficient consideration of GBM diversity. [12] Notably, there remains a critical gap in the systematic evaluation of TMZ-based combinations across a broad panel of genetically and phenotypically distinct GBM models, which is essential for identifying clinically translatable strategies.

Another critical consideration in designing combination regimens for GBM is ensuring that each therapeutic reaches the tumor site at a pharmacologically effective dose. [13] Unlike many targeted therapies, TMZ readily crosses the blood–brain barrier (BBB) following oral administration, making it a strong candidate for systemic use in combination strategies. [14] One approach to enhance the efficacy of TMZ is to locally deliver a synergistic agent at the time of tumor resection, thereby bypassing the BBB altogether. Currently, Gliadel^®^ is the only FDA-approved platform for localized GBM therapy. [15] It consists of a biodegradable polyanhydride copolymer (Polifeprosan 20) loaded with the DNA alkylating agent, carmustine (BCNU), administered intraoperatively. While preclinical studies were promising, Gliadel^®^ has not demonstrated a clear survival advantage over TMZ, likely due to its rapid burst release kinetics and limited drug diffusion into surrounding brain tissue. [16] Although modest benefit has been observed when Gliadel^®^ is combined with standard therapy, this dual-alkylating strategy (TMZ + BCNU) lacks molecular precision, failing to account for tumor subtype and, instead, takes a one-size-fits-all approach that ignores GBM’s complex heterogeneity. [17] Additionally, the polyanhydride matrix has been associated with increased neurotoxicity and peritumoral edema, attributed to both the high local BCNU concentrations and inflammatory responses triggered by its degradation byproducts—underscoring the need for more controlled, tunable platforms for safe localized drug delivery. [15,16]

Biodegradable electrospun polymer formulations have shown significant promise for localized GBM treatment by enabling site-specific, sustained drug delivery with minimal systemic exposure. These systems can be engineered to match the therapeutic needs of the brain microenvironment, offering controlled release profiles and compatibility with soft tissue. [18–20] Acetalated dextran (Ace-DEX) is a biocompatible and biodegradable polymer that offers a broad range of tunable and pH-neutral degradation rates, making it well-suited for such applications. [21] When drugs are incorporated into electrospun Ace-DEX nanofibers, the resulting matrix provides controlled and sustained release that improves local drug exposure at the target site when compared to conventional polymer systems. [22] These scaffolds are thin, flexible, and compatible with brain tissue, and they degrade into non-toxic byproducts, supporting their use as an advanced platform for localized therapy in GBM. [23]

In this study, we systematically evaluated 9 drugs known to target pathways identified in TCGA analyses in combination with TMZ across a panel of molecularly diverse GBM cell lines. The most effective candidate was then electrospun into an Ace-DEX scaffold to enable local delivery at the time of tumor resection. We tested this treatment in a clinically relevant and patient-derived mouse model of GBM that included both tumor resection and systemic TMZ chemotherapy. The results highlight the therapeutic promise and clinical relevance of this combination strategy for improving outcomes in this highly aggressive disease.

## 2. Material & methods

### 2.1. Drugs

A total of 10 drugs were obtained for in vitro drug screens. Drugs were ≥ 98 % purity confirmed by HPLC and acquired from MedKoo Biosciences (CAS#85622–93–1 temozolomide, TMZ – Durham, NC); Biosynth (CAS#284461–73–0 sorafenib, SFN – Louisville, KY); BOC Sciences (CAS#944396–07–0 buparlisib, BUP – Shirley, NY); LC Laboratories (CAS#183319–69–9 erlotinib hydrochloride and CAS#183321–74–6 free base, ERL; CAS#1195765–45–7 dabrafenib, DBR; CAS#871700–17–3 trametinib, TRM; and CAS#159351–69–6 everolimus, EVR – Woburn, MA); MedChemExpress (CAS#1229705–06–09 idasanutlin, IDSN – Monmouth Junction, NJ); and Tocris Biosciences (CAS#72835–26–8 MIRA-1, MIRA; CAS#1211441–98–3 ribociclib, RBC – Bristol, United Kingdom).

### 2.2. Cell lines and culture conditions

A total of 11 glioma cell lines were included for vitro drug combination assessments. Classically used human (Hu) glioma cells, including LN18 (#CRL-2610), LN229 (#CRL-2611), and U87 (#HTB-14), were purchased from American Type Culture Collection (ATCC - Gaithersburg, MD). U251 (Cat.#09063001) was acquired from the European Collection of Authenticated Cell Cultures (ECACC, Porton Down, UK). The murine (Mu) cell line CT2A, which expresses firefly luciferase (‘CT2A-Luc’), was purchased from Millipore Sigma (Burlington, MA, Cat.#SC195). The Mu cell lines GL261 and TRP were a gift from the Hingtgen Lab at UNC. [24] Human patient-derived (PDx) models including U3013PN, U3017CL, and U3031MS were purchased through the Human Glioma Cell Culture (HGCC) resource at Uppsala University (Uppsala, Sweden). [25] The human patient-derived GBM8 line was included as a gift from Dr. Hiroaki Wakimoto (Massachusetts General Hospital, Boston, MA).

Adherent cells including LN18, LN229, U87, U251, CT2A, GL261, and TRP were cultured in high-glucose Dulbecco’s Modified Eagle Medium (DMEM, ThermoFisher – Gaithersburg, MD) supplemented with 10 % heat-inactivated fetal bovine serum (FBS) and 1 % penicillin/streptomycin (Corning – Corning, NY). The HGCC PDx cells were cultured in DMEM/F12 glutamax and Neurobasal medium (1:1 vol.) supplemented with 2 % B27, 1 % N2, 0.5 % antibiotic-antimycotic (ThermoFisher), 10 ng/mL human epidermal growth factor (EGF) and 10 ng/mL fibroblast growth factor (FGF) (PeproTech – Cranbury, NJ). These cells were maintained and treated as adherent cultures by pre-treating T-75 culture flasks and 96-well plates with 10 μg/mL of polyornithine (Sigma-Aldrich – St. Louis, MO) in cell culture grade water for 3 h at room temperature or 4 °C overnight. Flasks and plates were then washed twice with phosphate-buffered saline (PBS, ThermoFisher), coated with 10 μg/mL laminin (Sigma-Aldrich) in PBS, and incubated for 30 min at 37 °C. The PDx GBM8 cells were cultured and treated as spheroids in neurobasal medium with 2 % B27, 1.5 % l-glutamine (ThermoFisher), 0.5 % N2, 0.5 % antibiotic-antimycotic, 2 μg/mL heparin salt (Sigma-Aldrich), and 20 ng/mL of EGF and FGF. All cells were determined mycoplasma-free with a mycoplasma detection kit (ThermoFisher) prior to use.

### 2.3. In vitro drug combinations and synergy quantification

For each cell line, five concentrations spanning the full dose-response curve of each drug were identified from the individual drug assessments and used for subsequent drug combination studies. Nine unique drug combinations consisting of TMZ and one of the other nine targeted therapies were assessed both individually and in combination, resulting in a 6 × 6 dose-response treatment matrix. In vitro combinations were screened in triplicate as described previously for the individual drugs and repeated for all 11 cell lines resulting in 99 drug combination experiments. Viability data was fit to a 4-parameter log-mean (4PL) trendline and was used as an input parameter for quantifying synergy.

The degree of synergy from the 99 combination screens was quantified using the SynergyFinder+ web-application. [26] The zero-interaction potency (ZIP) method was chosen as the primary metric for scoring synergistic interactions across the 5 × 5 drug combination-dosing matrix, resulting in 25 ZIP scores (δ) for each of the combinations and 1 ‘summary ZIP score’ (Δ) averaged across the combination matrix. The computed score quantifies the percent deviation of the observed effects from predicted zero-interaction additive effects based on the individual dose-response curves. These effects were qualified as antagonistic when −100 ≤ δ(%) < −1, additive when −1 ≤ δ(%) ≤ 1, and synergistic when 1 < δ(%) ≤ 100 To summarize the combination effects of a 2-drug combination in a single cell line and across the dose-response landscape, all 25 δ scores were averaged to generate a summary ZIP score (Δ). This Δ score was used to identify synergistic drug combinations for each cell line using the same designations for antagonism, additive, and synergism as the δ score. See Supplemental Eqs. 1–2 for ZIP synergy quantifications.

The adverse toxicity of selected synergistic drug combinations was assessed in non-tumor brain cells. Brain tissue was harvested from healthy C57BL/6 female mice, homogenized in Hank’s Buffer Salt Solution (HBSS, Corning), and isolated using 30 % Percol (ThermoFisher). The primary brain cells were seeded in a 96-well plate at 75,000 cells/well, immediately treated with a 5 × 5 dosing matrix of ERL and TMZ and incubated at 37 °C for 24 h. To assess viability, cells were stained for 15 min at room temperature with Fixable Viability Dye eFluor^™^ 506 (Fisher), diluted 1:1000 in FACS buffer (PBS + 2 % FBS). The stained cells were washed with FACS, fixed in 1 % paraformaldehyde (PFA, ThermoFisher) for 30 min on ice, and resuspended in FACS before performing flow cytometry (Fortessa). Flow data analysis was performed using FlowJo (Becton Dickinson). The frequency of viable cells for each treatment condition was normalized to the untreated control to determine the percent viability following treatment. ZIP synergy effects were quantified using the viability data with the same methods previously described.

### 2.4. Ace-DEX polymer synthesis

For the synthesis of acetalated dextran (Ace-DEX), dextran derived from *Leuconostoc* spp. (450–650 kDa); anhydrous dimethyl sulfoxide (DMSO); pyridinium *p*-toluenesulfonate (PPTS); and triethylamine (TEA) were purchased from Sigma-Aldrich. The acid catalyst, 2-ethoxypropene, was purchased from Matrix Scientific (Elgin, SC). Ace-DEX was synthesized following methods similar to those previously described. [21] In summary, dextran was first freeze-dried before being dissolved in DMSO. PPTS served as an acid catalyst to initiate the reaction with 2-ethoxypropene under anhydrous conditions. The polymer’s relative cyclic-to-acyclic acetal coverage (%CAC) was controlled by adjusting the reaction duration, which inversely correlates to the polymer degradation rate. The reaction was quenched using TEA. The synthesized Ace-DEX was isolated and purified using a liquid-liquid extraction method with ethyl acetate and Milli-Q^®^ water in a 2:1 (*v*/v) ratio, followed by separation into the organic phase. The solvent was removed through rotary evaporation, after which the polymer was redissolved in ethanol. To further purify the material, the solubilized Ace-DEX was gradually added to basic water (0.04 % TEA in Milli-Q^®^ water), vacuum filtered, freeze-dried, and stored at −20 °C until needed. Proton nuclear magnetic resonance (^1^H NMR, Varian Inova 400 MHz) analysis was used to assess the % CAC (Supplemental Fig. S1).

### 2.5. Electrospinning Ace-DEX scaffolds and in vitro characterization

Erlotinib (free-base, ERL) was encapsulated in Ace-DEX via electrospinning, as previously described. [23,27,28] Briefly, Ace-DEX was dissolved at 200 mg/mL in a 60:40 (% *v*/v) solution of hexafluoro-2-propanol (HFIP) and 1-butanol (Sigma-Aldrich) with 1 % (v) TEA. ERL was added to the dissolved polymer such that the final concentration of drug was 22.2 mg/mL (or theoretical loading of 10 % by weight). Using a blunt 21 G needle, the prepared Ace-DEX + ERL solution was loaded into a glass Hamilton syringe and positioned opposite a collection plate. A voltage differential of −7.5 kV at the collection plate and + 7.5 kV at the syringe needle was maintained across a 13 cm working distance. The solution was dispensed from the syringe at a steady flow rate of 1 mL/h, resulting in the formation of solid, randomly oriented drug-loaded polymeric fibers on the collection plate. ERL was encapsulated within two separate scaffolds using Ace-DEX polymers of 47 % CAC and 50 % CAC, respectively. To ensure consistency for the in vitro and in vivo studies, samples were taken from the same bulk scaffold. Fiber morphology was evaluated by scanning electron microscope (SEM, Hitachi S-4700 Cold Cathode Field Emission; UNC CHANL) at 2 kV on samples mounted onto a palladium sputter-coated stub. Fiber diameter was determined using ImageJ software, with measurements reported as the mean ± standard deviation based on at least 30 recorded values (*n* ≥ 30). Experimental drug loading was determined in triplicate where 0.6–1.2 mg samples of the scaffold were dissolved in DMSO at 1 mg/mL and detected for ERL concentration via absorbance at 330 nm against a standard curve. Supplemental Eq. 3 was used to quantify the percent weight loading of ERL in each scaffold. Scaffolds were stored at −20 °C until further use.

In vitro studies for scaffold mass retention and drug release were conducted in triplicate for each time point. Approximately 1 mg scaffold samples were incubated at 37 °C in an equivalent volume of PBS (for 1 mg/mL) on a shaker plate to mimic physiologic conditions. At designated timepoints, scaffold samples were removed from PBS, washed in basic water, and lyophilized. Dried samples were reweighed to determine scaffold mass loss (Supplemental Eq. 4) and assessed for drug retention as described above. Drug released over time was quantified using Eq. 1:

(1) $$\%\text{Drug}\;\text{Released}=\left(1-\frac{\text{Retained}\;\text{Drug}\;\text{Concentration}\left(t\right)}{\text{Drug}\;\text{Concentration}\left(t=0\right)}\right)\times 100\%$$

### 2.6. In vivo studies

Athymic nude female mice (Crl:NU(NCr)-Foxn1nu) were sourced from the Animal Studies Core at the University of North Carolina at Chapel Hill (UNC-CH) and age-matched for all in vivo experiments. The study received approval from the UNC-CH Animal Care and Use Committees, ensuring compliance with guidelines established by the National Institutes of Health Guide for the Care and Use of Laboratory Animals and the American Veterinary Medical Association. Mice were anesthetized using vaporized isoflurane for all surgical procedures. To manage pain, bupivicane (1 mg/kg) was administered at the incision site, and subcutaneous meloxicam (5 mg/kg) was given before surgery and continued once daily for three days post-operation. Humane endpoints were predefined and applied consistently across all studies, and mice were euthanized when humane endpoints were reached; ≥20 % weight loss or if they exhibited signs of distress or gait abnormalities. All animals were included in the final analysis of the respective studies. Mice with unsuccessful tumor implantation or surgical complications would have been excluded based on predefined criteria, though no such exclusions occurred.

### 2.7. Pharmacokinetics

The pharmacokinetics of erlotinib released from intracranially implanted 47 % CAC Ace-ERL scaffolds (100 μg total erlotinib) was evaluated in non–tumor-bearing female athymic nude mice (*n* = 12). Mice were randomly assigned to terminal collection at 1 h, 1, 5, or 7 days post-implantation (*n* = 3 per time point). At each time point, mice were euthanized, and blood and brain tissues were collected. The remaining scaffold was recovered, lyophilized, dissolved in DMSO, and analyzed by UV absorbance to quantify residual drug content (Eq. 1). Randomization and consistent handling procedures were applied across timepoints; no blinding was performed as outcome measures were objective.

Harvested brain tissue and plasma were snap frozen. Brain tissue was homogenized in PBS using 1.4 mm porcelain homogenization beads and a VWR bead mill homogenizer. Homogenate was diluted in PBS for a 4× dilution then further diluted 2× in plasma for a total 8× dilution. Erlotinib was extracted from tissue and plasma using protein precipitation in acetonitrile with 0.1 % formic acid, centrifuged, then supernatant was diluted 1:5 in water with 0.1 % formic acid. This mixture was evaluated by LC-MS/MS to quantify the amount of erlotinib in each brain or plasma sample. The lower limit of quantification was 4 ng of erlotinib per gram of tissue (which is equivalent to 0.5 ng erlotinib per 1 mL homogenate) for brain and 0.5 ng per mL for plasma.

A Waters XBridge BEH C18 column (SKU:186006029 – Milford, MA) was utilized, operating at *T* = 40°*C*. Mobile Phase A consisted of water with 0.1 % formic acid (100:0.1, *v*/v). Mobile Phase B consisted of acetonitrile with 0.1 % formic acid (100:0.1, v/v). The mobile phase gradient profile was: 0.0–0.75min at 10 % B. From 0.75 to 2.50 min concentration of B changed from 10 % to 100 %. 100 % B was held from 2.50 to 3.50 min. The gradient changed from 100 % to 10 % B from 3.50 to 3.55 min and was held at 10 % B from 3.55 to 4.80 min. Needle Wash Solvent 1 consisted of acetonitrile with 0.1 % formic acid (100:0.1, v/v). Needle Wash Solvent 2 consisted of water with 0.1 % formic acid (100:0.1, v/v). The flow rate was set at 0.300mL/min for a total run time of this assay of 4.8min.

The electrospray source was utilized in the positive ion mode and the tandem mass spectrometer was operated with the spray voltage set at 3.0kV. The capillary temperature and vaporizer temperature were set at 232 °C and 248 °C, respectively. Quantification was conducted with the multiple reaction monitoring (MRM) mode using the ion transitions: *m*/*z* 394 > 278 for erlotinib and 400 > 278 for the internal standard erlotinib-d6. The collision energy for erlotinib was set at 29 eV.

### 2.8. Glioblastoma model for resection and recurrence

For in vivo efficacy studies, a mouse model for GBM resection and recurrence was used. In brief, 100,000 GBM8-mCh-FLuc cells in 2 μL PBS were injected into the right hemisphere of the brains of nude mice (2 mm lateral of bregma, 0.5 mm below dura) at a flowrate of 1 μL/min. Tumors were allowed to develop for 11 days and monitored for growth via bioluminescence (BLI, IVIS Spectra). Under fluorescent guidance, tumors were then partially resected before administering treatment. Vetbond tissue adhesive (3 M – St. Paul, MN) was used to close the surgical window, and mice were routinely monitored for weight loss and signs of pain.

### 2.9. In vivo TMZ pilot study

To determine the dose of TMZ needed for mimicking the marginal benefits of clinical TMZ in the standard of care, *n* = 17 mice bearing GBM8-mCh-Fluc tumors (as described above) received either no treatment (*n* = 3) or a TMZ treatment regimen of 25 (*n* = 4), 10 (n = 4), or 5 mg/kg (n = 3) intraperitoneally (I.P.) on days 2, 4, 7, 9, and 11 after resection or 5 mg/kg I.P. (n = 3) on days 3, 5, 7, and 9. The optimum dose was identified by comparing median survival and tumor recurrence (via BLI) relative to untreated controls.

### 2.10. In vivo combination efficacy study

This study was designed to evaluate the therapeutic efficacy of local Ace-ERL scaffolds, systemic temozolomide (TMZ), and their combination in a mouse model of glioblastoma resection and recurrence (as described above). Sample sizes from *n* = 27 mice were selected based on expected survival trends from preliminary pilot studies and prior experience with this model, with the goal of observing meaningful differences in survival and tumor recurrence while minimizing animal use. Mice were randomly assigned to four groups—no treatment (“No Tx”, *n* = 6), Ace-ERL alone (“Ace-ERL”, *n* = 10), TMZ alone (“TMZ”, *n* = 7), or the combination of Ace-ERL and TMZ (“Combo”, *n* = 14)—with randomization stratified to ensure comparable average tumor sizes across groups based on pre-resection bioluminescence imaging (BLI).

At the time of surgical resection, mice receiving Ace-ERL treatment received a locally administered 47 % CAC Ace-ERL scaffold with an equivalent dose of 25 μg ERL (for an empirical 10 % wt./wt. ERL-loaded scaffold, 0.25 mg of Ace-ERL achieves 25 μg of ERL) in the tumor resection cavity. For mice also receiving the standard of care, the dose of 5 mg/kg I.P. TMZ on days 3, 5, 7, and 9 after resection was administered.

The primary outcomes were survival and tumor progression. Mice were routinely monitored for BLI and humane endpoints to track tumor recurrence and survival following resection surgery. Disease progression was defined as a 200-fold increase in tumor bioluminescent signal normalized to Day 1 and interpolated from a best-fit trendline of normalized bioluminescent signal vs. time for each mouse. Progression-Free Survival (PFS) models for additivity and highest single agent (HSA) were discerned from adjusted sample sizes (*n* = 14) for each individual therapy and using Eqs. 2 and 3 where PFS_Ace-ERL_, PFS_TMZ_, and PFS_No Tx_, is the time-matched % PFS of Ace-ERL alone, TMZ alone, and untreated mice respectively. Randomization and consistent handling procedures were applied across groups; no blinding was performed as outcome measures were primarily objective (% weight loss and tumor bioluminescence).


(2) $$PFS_{\text{Additivity}}=PFS_{Ace-ERL}+PFS_{TMZ}-PFS_{No\;Tx}$$

(3) $$PFS_{HSA}=\text{max}\left(PFS_{Ace-ERL},PFS_{TMZ}\right)$$

### 2.11. Statistical analysis

All in vitro studies were performed in triplicate at each concentration or time point with data points as mean ± standard deviation. In vitro viability data and in vivo survival data were assessed for significance via ordinary one-way ANOVA with Tukey’s multiple comparisons and log-rank (Mantel-Cox) tests, respectively in GraphPad Prism with *P*-values of <0.05 considered statistically significant between groups. Statistical analysis for synergy scores were performed on the opensource SynergyFinder^+^ website (https://tangsoftwarelab.shinyapps.io/synergyfinder/_w_9cd59553/#!/). Observed in vivo combination effects were compared to the additivity and HSA models by the hazard ratio (HR) where an HR <1 indicates observed results were superior to the predicted model.

## 3. Results & discussion

### 3.1. Temozolomide response is variable among GBM models and targeted therapies showcase unique mechanisms to enhance standard of care treatment

**Fig. 1.**
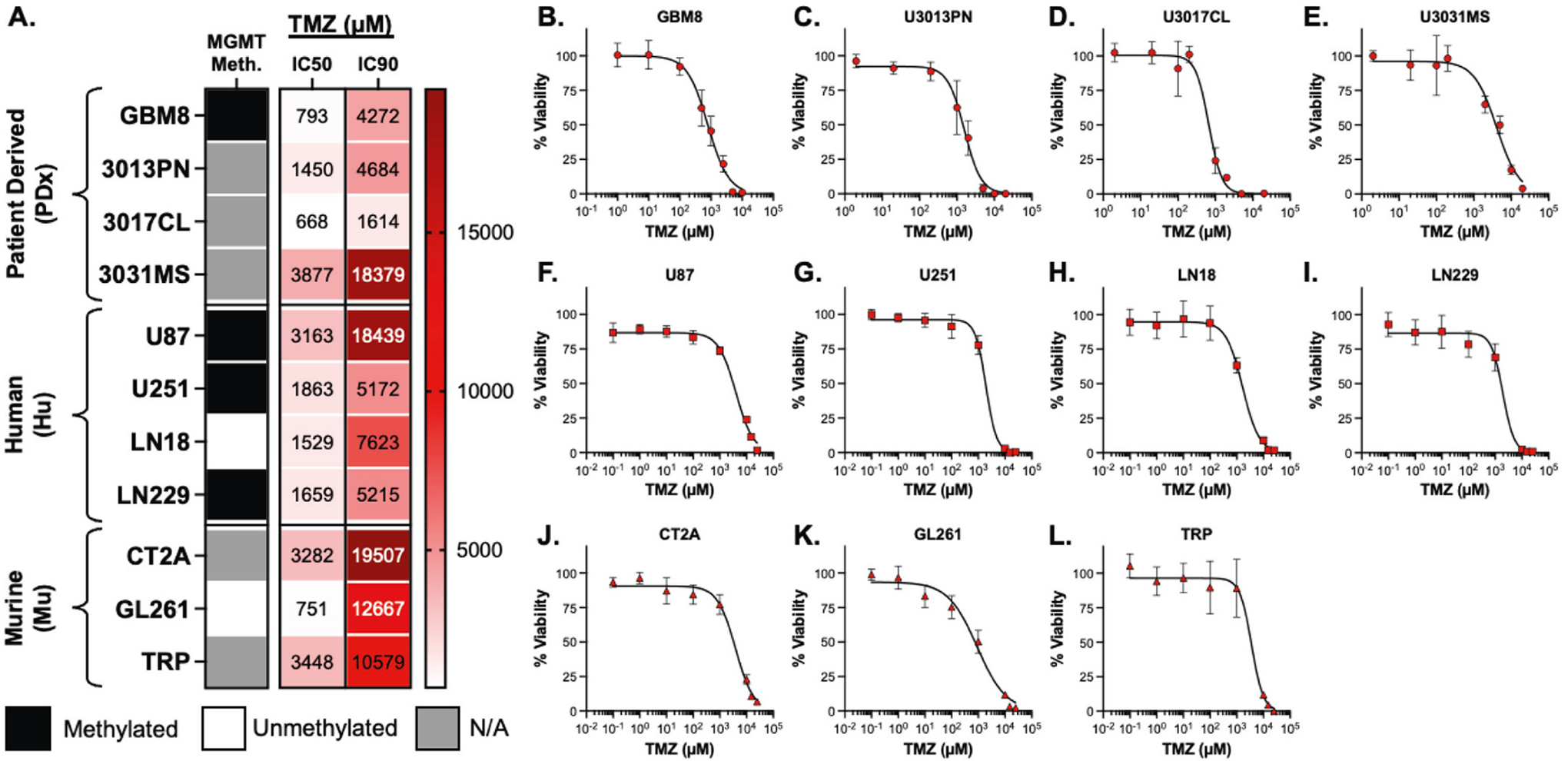
Panel of GBM cell lines treated with temozolomide, TMZ. (A) Heatmap of 11 cell lines, differentiated by model type, with respective methylation (meth.) status of O^6^-methylguanine-DNA methyltransferase (MGMT) and the TMZ inhibitory concentrations (IC) which achieved 50 % viability or 10 % viability (IC50 and IC90, respectively) in vitro. MGMT methylation status indicated as reported from literature (see Supplemental Table S1) and IC50/IC90 values were interpolated by a 4-parameter log-mean trendline from in vitro drug screening data. (B-L) Dose-response curves for TMZ in vitro for each cell including (B-E) patient-derived (PDx) cells, (F-I) human models (Hu), and (J-L) murine (Mu) models. Individual points are the mean ± standard deviation.

The panel of 11 glioblastoma (GBM) cell lines selected for this study includes four patient-derived xenografts (PDx), four human-derived models (Hu), and three murine models (Mu). The PDx models are distinguished from the Hu lines by their maintenance in serum-free, physiologically relevant culture conditions, which enhance their translational relevance. This panel spans a heterogeneous range of O^6^-methylguanine-DNA methyltransferase (MGMT) promoter methylation statuses, as compiled from a literature review (Fig. 1A, Supplemental Table S1).

To evaluate GBM model responses to temozolomide (TMZ), we assessed cell viability following a 48-h in vitro treatment. A broad range of TMZ sensitivities was observed across the panel, reflecting the variable therapeutic response commonly seen in GBM patients treated with standard of care chemotherapy. Notably, differences in TMZ potency were also evident within individual model types (PDx, Hu, Mu). Among the PDx models, the 50 % inhibitory TMZ concentrations (TMZ IC50, μM) ranged widely (3209 μM), with U3031MS showing the highest IC50 (3877 μM) and U3017CL the lowest (668 μM) (Fig. 1B–E). In contrast, the Hu models displayed the narrowest IC50 range (1634 μM), with all lines exhibiting values greater than 1500 μM (Fig. 1F–I). The Mu models showed a broader IC50 range (2697 μM), similar to the PDx group, but exhibited substantially weaker overall drug sensitivity, with 90 % inhibitory TMZ concentrations (TMZ IC90, μM) exceeding 10 mM in all cases (Fig. 1J–L). These findings underscore the importance of using a diverse set of GBM models for preclinical evaluation of treatment efficacy. In particular, the inclusion of multiple PDx models better captures the variability observed in patient tumors and provides a more clinically relevant assessment compared to conventional Hu or Mu models.

When comparing in vitro TMZ sensitivity with literature-reported MGMT promoter methylation status, we observed little correlation between methylation and cell-line–specific drug response. Although MGMT expression data from the literature can be variable, only two cell lines—LN18 and GL261—were consistently reported as having unmethylated MGMT, which would typically suggest reduced TMZ sensitivity. [29,30] However, both lines exhibited the lowest IC50 values within their respective Hu and Mu model groups, contradicting expectations. Interestingly, GL261 also displayed the highest fold-increase from the IC50 to the IC90 (16.9), which was notably greater than the average fold-increase observed across MGMT-methylated cell lines (4.3). This elevated change implies a shallow dose-response curve, indicative of a weaker overall drug effect despite low initial sensitivity. The patient-derived xenograft (PDx) cell line GBM8, which is reported to have a methylated MGMT promoter, exhibited a similarly elevated IC50 to IC90 fold-change of 5.4. [31] While its low IC50 is consistent with expected TMZ sensitivity, the higher IC90 suggests the presence of heterogeneous subpopulations that require higher drug concentrations for complete inhibition. [32] This may more accurately reflect the clinical complexity of GBM, as neurosphere cultures derived from PDx models can encompass both TMZ-sensitive and TMZ-resistant cell populations. [33] These findings underscore the limitations of using MGMT promoter methylation status as a standalone predictive biomarker for TMZ response. Given the variability in detection methods and the plasticity of methylation status over time or in culture, careful in vitro and in vivo evaluation remains essential to reliably predict TMZ efficacy. [34]

**Fig. 2.**
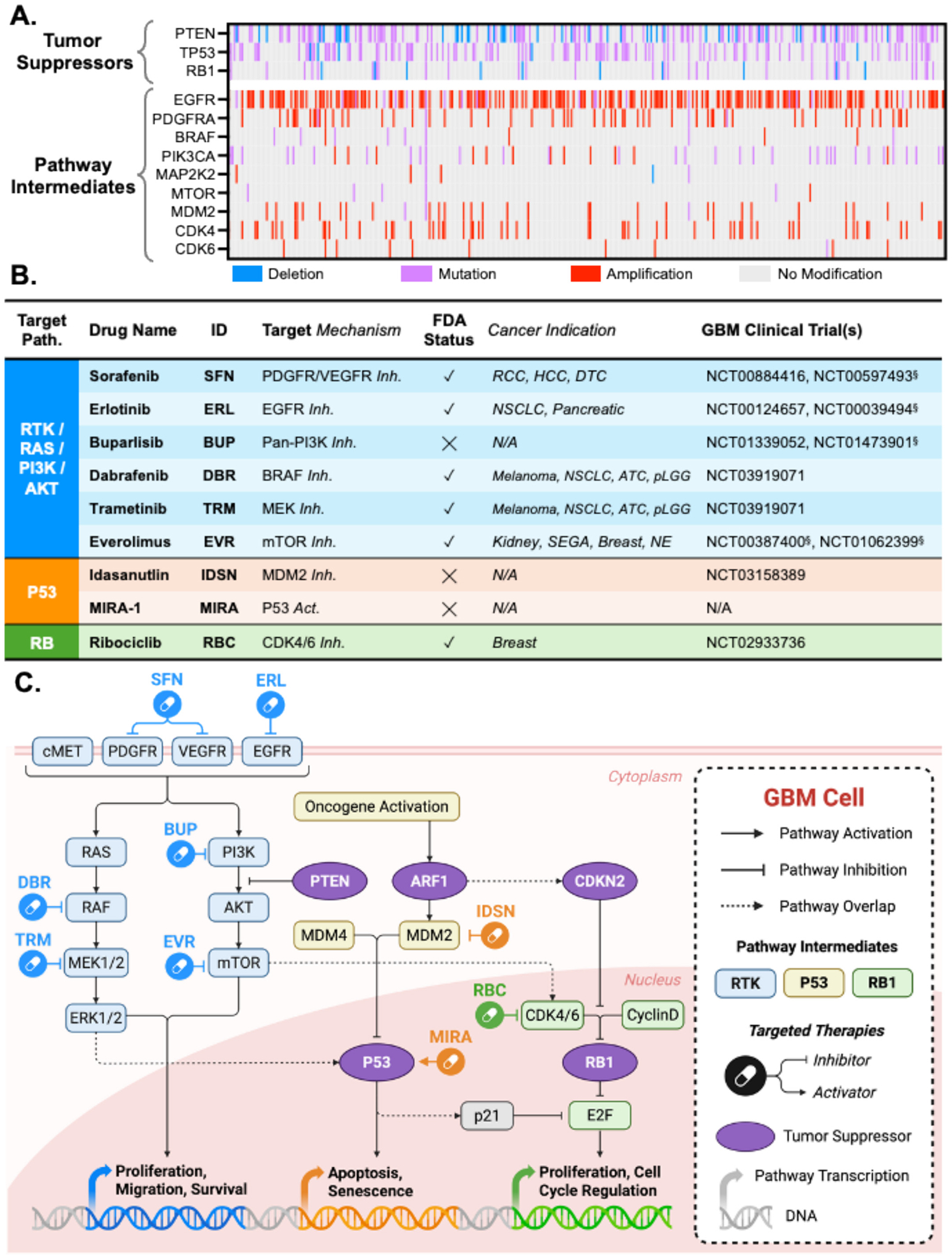
Curated GBM Drug Library chosen for assessing combination effects with TMZ. (A) Relevant gene expressions for *n* = 513 patients from the Cancer Genome Atlas (TCGA) project. (B) Table of 9 targeted therapies, grouped by target pathway (path.), and included in the prospective study. Mechanisms of drugs are denoted as inhibitors (Inh.) or activators (Act.) of the indicated target or pathway intermediate. FDA approval status (✓ = approved, × = not approved) for various cancer treatments (RCC = renal cell carcinoma, HCC = hepatocellular carcinoma, DTC = differentiated thyroid carcinoma, NSCLC = non-small cell lung cancer, ATC = anaplastic thyroid cancer, pLGG = pediatric low-grade glioma, SEGA = subependymal giant cell astrocytoma, NE = neuroendocrine). National clinical trial (NCT) numbers of drug evaluated for GBM. NCT^§^ = drug + temozolomide. (C) Schematic of pathways commonly mutated in GBM with overlaid drugs from (A) on the respective pathway target. Pathway intermediates and transcription are differentiated by colour from left to right (RTK/RAS/PI3K/AKT in blue, P53 in orange, and RB1 in green).

With the established heterogeneity of the chosen GBM cell lines and the evidence highlighted in the Cancer Genome Atlas (TCGA) project, the curated drug library was established to offer unique and GBM-relevant mechanisms for observing a potential benefit when combined with TMZ (Fig. 2A–B). The nine selected therapies included are small molecule drugs targeting intermediates within the three most frequently mutated pathways in GBM tumors: RTK/RAS/PI3K/AKT (RTK), P53, and RB1 (Fig. 2C). These pathways are not only prevalent in GBM, but they are commonly mutated in many other malignancies as they are responsible for a variety of metabolic and cell-cycle regulating processes such as proliferation, migration, and regulating apoptosis. [35] Targeting these aberrations in addition to the standard of care chemotherapy offers unique mechanisms for affording synergistic effects while reducing the chance of developing therapeutic resistance. [36]

Several of the selected targeted therapies, including sorafenib (SFN), erlotinib (ERL), and everolimus (EVR), are FDA-approved for the treatment of other malignancies and have also been evaluated clinically in combination with TMZ for GBM. [37–40] However, these combination strategies have shown limited success when compared to the standard therapy alone. This is often attributed to suboptimal trial design, dose-limiting toxicities, and a lack of patient stratification by molecular profiling. [12] To improve the likelihood of clinical translation, drug combinations should be rigorously evaluated across molecularly diverse GBM models to ensure consistent dual-agent efficacy in both in vitro and physiologically-relevant in vivo settings. Of note, combination trials including TMZ with SFN (NCT00597493), ERL (NCT00039494), and EVR (NCT01062399) have historically been evaluated via oral delivery of the therapeutic agents, highlighting a common feature of previously failed attempts. [37–39] Future strategies may benefit from localized drug delivery approaches that maximize therapeutic exposure at the tumor site and overcome the pharmacokinetic limitations associated with systemic administration.

### 3.2. In vitro synergy scores for drug combinations reveal model-dependent differences in therapeutic response, with TMZ + erlotinib demonstrating the most consistent efficacy across all models

For combination therapies to be successful, they must be supported by robust preclinical evidence and a mechanistic rationale. [41] In vitro screening approaches often evaluate drug interactions using reference models such as the highest single agent (HSA), Loewe additivity, and Bliss independence. [42,43] These models compare observed combination effects to theoretical expectations to classify combinations as antagonistic (less effective), additive (equal to the sum of individual effects), or synergistic (more effective). While widely used in vitro, they face limitations when predicting in vivo response. The zero-interaction potency (ZIP) model, however, offers a more comprehensive framework by evaluating dose-response synergy across a full matrix of combinations, improving translatability to in vivo settings. [26]

**Fig. 3.**
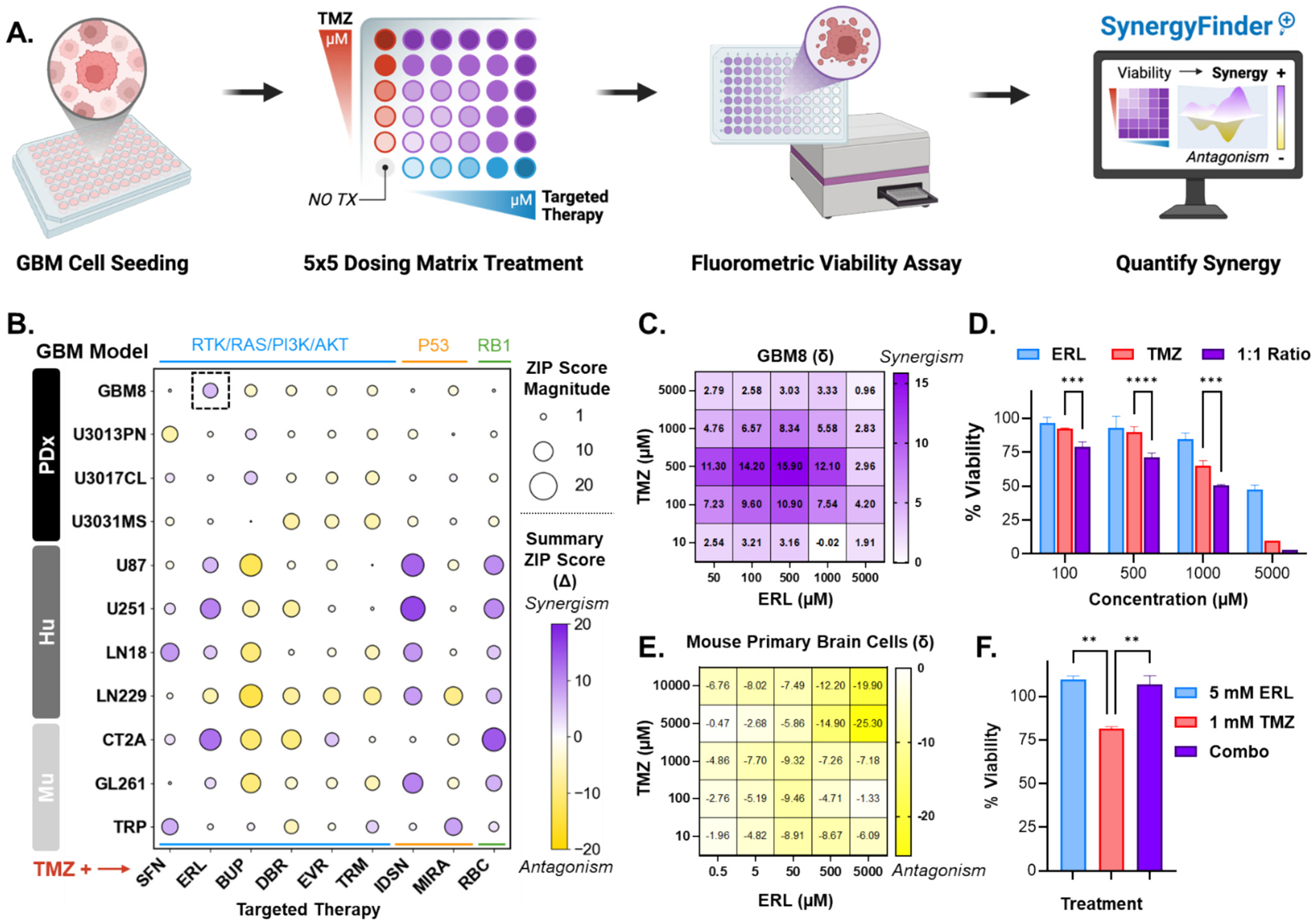
In vitro synergy screen results. (A) Method for quantifying combination effects of targeted therapy + TMZ in vitro. GBM cells are first seeded in a 96-well plate format. Cells are then treated with a 5 × 5 increasing dose matrix of the two drugs before assessing viability via fluorometric assay. The viability data is then used to calculate synergy scores across the dosing matrix using SynergyFinder^+^ interface. (B) Bubble plot showing the summary Zero-Interaction Potency (ZIP) Synergy results for TMZ + each targeted therapy (x-axis, grouped by target pathway) when screened in the GBM Cell Lines (y-axis, grouped by model type). The shading of the bubble corresponds to the value of the summary ZIP score (Δ) where yellow is a negative or antagonistic score, white is 0 or additive, and purple is positive or synergistic. The size of the bubble corresponds to the magnitude of that score. (C) Heatmap for the matrix of ZIP scores (δ) afforded by each of the 25 combinations of TMZ and erlotinib (ERL) in the GBM8 cell line, corresponding to the summary ZIP score shown in (A) with the dashed-line box. All 25 δs are averaged to get Δ. (D) Dose-response (% viability) for ERL, TMZ, and ERL + TMZ at a 1:1 M ratio. (E) Heatmap for the matrix of ZIP scores (δ) when TMZ and ERL were screened in mouse primary brain cells. (F) Corresponding % viability in mouse primary brain cells for 5 mM ERL, 1 mM TMZ, and the combination of 5 mM ERL + 1 mM TMZ. Error bars are ± standard deviation. **p < 0.01, ***p < 0.001, ****p < 0.0001 by ordinary one-way ANOVA with Tukey’s multiple comparisons test.

To evaluate the potential synergy between TMZ and each of the 9 targeted therapies, we conducted in vitro screening across the panel of 11 GBM cell lines (Fig. 3A). Drug interactions were quantified using the ZIP model, which calculates a synergy score (δ) from cell viability data obtained across a 5 × 5 combination-dose matrix. In this context, negative scores reflect antagonism, scores near zero indicate additive effects, and positive scores suggest synergistic interactions. A summary ZIP score (Δ) was then calculated as the average of all 25 δ values from each matrix. Across all combinations and cell lines, 99 summary ZIP scores were generated (Fig. 3B), ranging from the most antagonistic interaction—TMZ + buparlisib (BUP) in LN229 (Δ = −14.01)—to the most synergistic—TMZ + idasanutlin (IDSN) in U251 (Δ = 16.14). These findings highlight the variability in treatment response across GBM models, underscoring the challenge of identifying a universally effective therapy.

Beyond combination-specific effects, synergy scores also varied by GBM model type. PDx cell lines, for example, exhibited the lowest average magnitude of summary ZIP scores while achieving mostly additive responses to the TMZ combinations when compared to that of Hu and murine Mu models. Of the 9 combinations tested, TMZ + ERL and TMZ + IDSN performed best overall, yielding positive ZIP scores in 9 of 11 cell lines each. However, TMZ + IDSN was antagonistic in two PDx lines, suggesting limited translational relevance. In contrast, TMZ + ERL showed additivity (−1 ≤ Δ ≤ 1) in a single PDx line (U3013PN; Δ = −0.83) and achieved the highest synergy score out of all combinations screened in the PDx models (Δ = 5.90 in GBM8). Given this result and the prevalence of EGFR alterations in GBM, we selected ERL for further evaluation in combination therapy with the clinically relevant and PDx model, GBM8.

The ZIP model also allows identification of optimal dosing regions for achieving maximum synergy. As shown in the in vitro results with GBM8, nearly all 25 combinations of TMZ + ERL across the 5 × 5 matrix produced positive δ scores (Fig. 3C). At combinations where TMZ:ERL were dosed at a 1:1 M ratio, the viability was significantly reduced when compared to monotherapies at their equivalent doses (Fig. 3D, Supplemental Fig. S2A). Notably, GBM8 cells, along with all other PDx models, harbor EGFR amplification, which may explain the poor response to ERL alone but enhanced sensitivity in combination with TMZ (Supplemental Table S2). [44]

Although TMZ + ERL synergy has been previously reported and explored in clinical trials, outcomes have remained poor due, in part, to systemic toxicities of orally dosed ERL. [45–47] To evaluate the safety and local effects of ERL, we tested TMZ + ERL in cells isolated from healthy brain tissue of C57BL/6 mice. Both individual drugs and all 25 combinations exhibited minimal toxicity (Supplemental Fig. S2B). Moreover, all combinations showed antagonistic ZIP scores in normal brain tissue (Δ = −7.75), and IC50-level doses achieved in GBM8 (1 mM TMZ and 5 mM ERL) maintained ≥75 % viability in healthy tissue (Fig. 3E–F). These findings support the potential of local ERL delivery to achieve enhanced activity against GBM while sparing off-target ERL toxicities when it is combined with TMZ.

### 3.3. Electrospinning erlotinib-loaded Ace-DEX scaffolds affords a platform for controlled release of encapsulated drug with favorable pharmacokinetics following interstitial delivery

As previously discussed, prior combination trials of ERL with TMZ have proven to be insufficient in outperforming the standard of care alone. High doses of oral ERL (>200 mg/day) are needed to achieve therapeutically effective drug concentrations at the tumor site which has commonly been associated to systemic toxicities and adverse effects like skin rash. [48] Although clinical pharmacokinetic studies have found ERL to penetrate the BBB and reach brain tumors, the overall drug exposure at the target site is thought to be insufficient for achieving the desired effect. [49] Furthermore, daily oral dosing is needed to achieve consistent treatment to residual tumor cells, harboring increased risk for adverse systemic toxicities. Therefore, a strategy which implements controlled and local delivery of ERL directly at the site of the tumor is needed to maximize drug exposure while minimizing off-target toxicities.

**Fig. 4.**
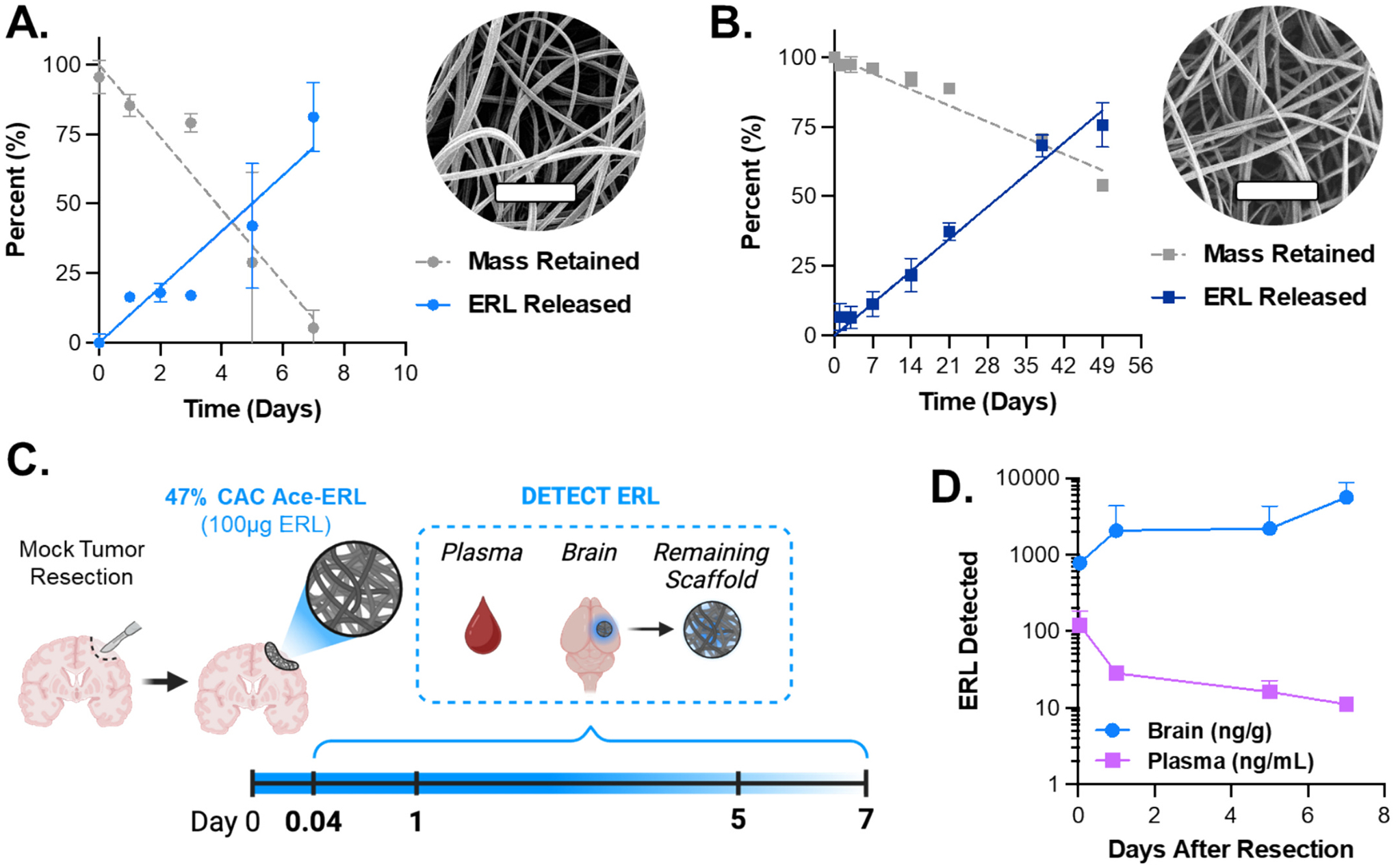
Ace-ERL provides controlled release of ERL and higher drug accumulation in the brain following interstitial delivery. (A-B) In vitro kinetics of drug release and mass retention of ERL-loaded electrospun scaffolds using Ace-DEX (Ace-ERL) with (A) 47 % cyclic acetal coverage (CAC) and (B) 50 % CAC polymer. Inlet images are representative scanning electron microscopy (SEM) photographs with scale bar = 10 μm. Error bars are ± standard deviation of time-matched replicates (*n* ≥ 3) in phosphate buffered saline (PBS, 37 °C pH 7.4). (C) Timeline for pharmacokinetic study with a 100 μg dose of ERL in 47 % CAC Ace-ERL scaffolds. Nude mice were first given mock resections followed by the local administration of Ace-ERL scaffolds. After 1 h, 1, 5, and 7 days, groups of *n* = 3 mice were euthanized. Any remaining scaffold was removed from the brain and collected tissues (plasma and brain) were assessed for ERL content via HPLC-MS. (D) Results from PK study for ERL detected in the brain (ng ERL/g tissue) and plasma (ng ERL/mL plasma). Data points are mean ± standard deviation.

To support the development of a localized therapy compatible with the GBM standard of care and demonstrate tunability in drug release rates, ERL was encapsulated at a theoretical loading of 10 % (wt./wt.) in electrospun acetalated dextran (Ace-DEX) scaffolds using two different polymer formulations (Fig. 4A–B). This approach encapsulates drug into thin, flexible, and biodegradable nanofiber matrices with drug release kinetics inversely related to the cyclic-to-acyclic acetal coverage (%CAC) of Ace-DEX (Supplemental Fig. S1). The %CAC determines the degradation rate of Ace-DEX where a lower %CAC yields faster degradation rates than a higher %CAC, which in turn correlates with drug release. [22] We see this trend in our two formulations of scaffolds, with 50 % of ERL released by day 5 for the 47 % CAC scaffold, compared to day 30 with the 50 % CAC scaffold (Table 1). Given the rapid progression of recurrent GBM in vivo, early drug release is critical during the initial post-resection period when tumor burden is low and standard therapies are oftentimes most effective. [50] Therefore, the 47 % CAC formulation was selected for further investigation and is referred to as Ace-ERL throughout the remainder of this study.

To evaluate ERL distribution following interstitial delivery, pharmacokinetic (PK) studies were conducted by placing Ace-ERL into a resection cavity in the brain of non-tumor bearing mice (Fig. 4C). Detected ERL levels and the area under the curve (AUC) in brain tissue remained substantially higher than in the plasma at all measured time points, with concentrations peaking after day 7, suggesting localized drug accumulation (brain AUC = 17,670 ± 7393 ng·day/g tissue, Fig. 4D). In contrast, plasma ERL peaked at 1 h and steadily declined thereafter (plasma AUC = 186.7 ± 34.57 ng·day/mL plasma). Notably, the sustained local release of ERL from Ace-ERL resulted in a 94.6(±43.3)-fold increase of ERL AUC in the brain relative to plasma, suggesting enhanced brain retention following the continuous Ace-DEX degradation. In contrast, the Gliadel^®^ wafer has shown to elicit rapid burst release of carmustine (BCNU) from the polyanhydride wafer in vivo with over 70 % of drug released in the first 24 h and resulting in decreasing interstitial BCNU concentrations after day 1. [51] Furthermore, reports of local tissue damage and toxicity have been reported for rigid, polyanhydride-based platforms like Gliadel^®^. [15,52] As we have shown previously, our Ace-DEX scaffolds, when implanted into the resection cavity, did not produce significant local toxicity days following treatment, as confirmed by histological analysis of the surrounding brain tissue. [22,23]

The remaining scaffold at each timepoint in the pharmacokinetic study was recovered, lyophilized, and analyzed by UV absorbance to quantify residual drug content (Eq. 1). Authors noted that the mass of the collected scaffold decreased significantly over time with very little remaining by day 7 (data not shown) which was comparable to the observed scaffold degradation in vitro (Fig. 4A). Moreover, the drug release kinetics of Ace-ERL appear to translate from in vitro to in vivo assessment, validating continuous and well-tolerated controlled release (Supplemental Fig. S3A–B). Taken together, these findings demonstrate the potential of electrospun Ace-DEX scaffolds to not only deliver therapeutics locally but offer a mechanism for sustained drug exposure in brain tissue—facilitating enhanced local combination strategies when co-treated systemically with BBB-penetrant agents such as TMZ.

### 3.4. Localized delivery of Ace-ERL at the time of resection extends survival of mice bearing patient-derived GBM8 tumors and enhances the standard of care, TMZ

As surgical resection of GBM tumors is a hallmark of treatment for newly diagnosed GBM, it is important to consider the physiological implications of this standard of care when evaluating new intervention strategies for improved outcomes. For example, debulking primary tumors has been shown to cause brain edema, which can dramatically change drug diffusion throughout the parenchyma. [53,54] Additionally, GBM tumors can recur more aggressively following resection, which together, highlights key challenges to improve upon this first-line treatment. [55] However, many preclinical studies fail to incorporate tumor resection into in vivo efficacy studies, thus limiting the translatable relevance to clinical GBM. [56,57]

**Fig. 5.**
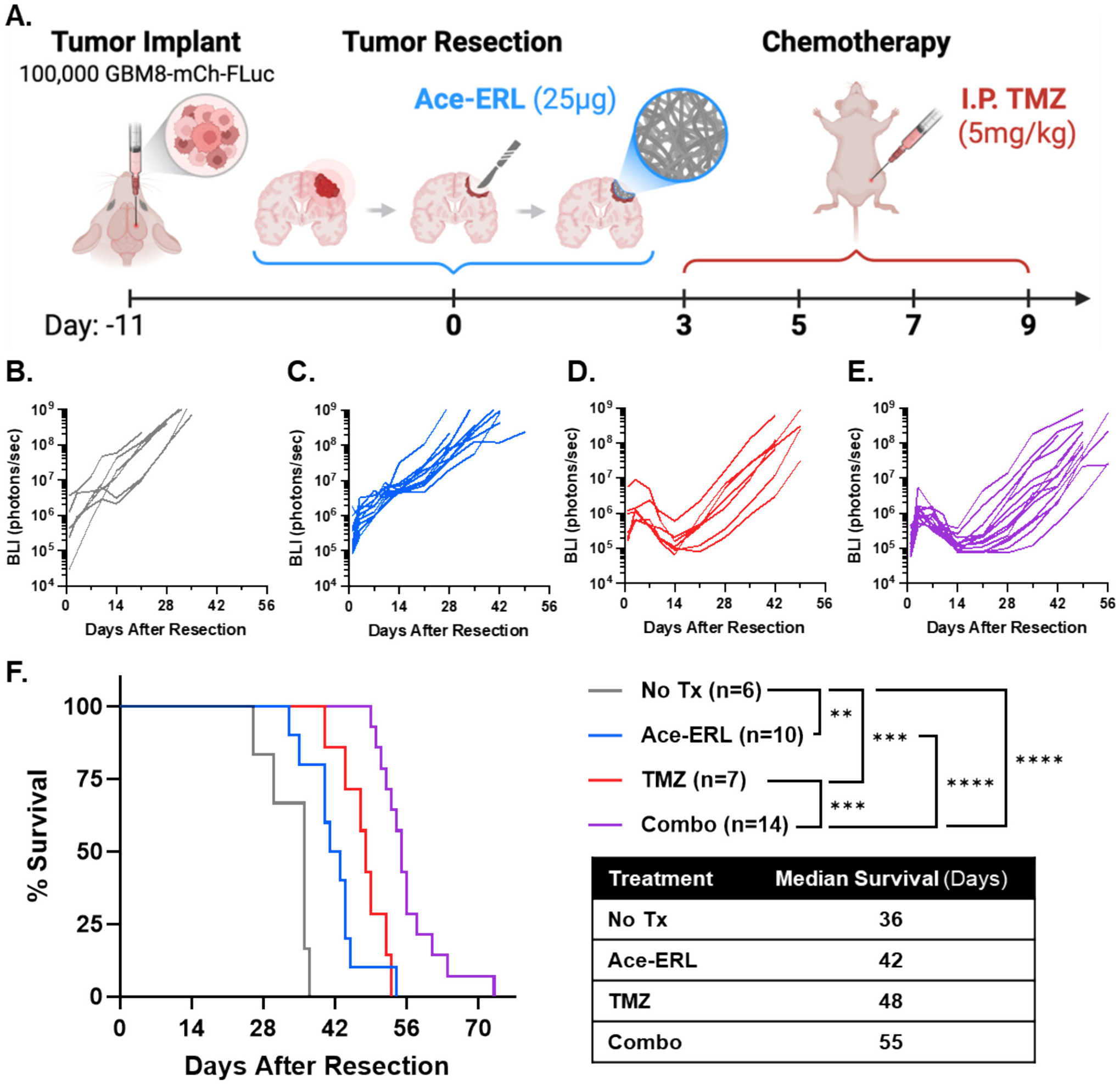
In vivo efficacy of Ace-ERL with the standard of care, TMZ. (A) Schematic of in vivo mouse model for evaluating treatment with GBM tumor resection and recurrence. 100,000 GBM8-mCh-FLuc cells were implanted orthotopically in the right hemisphere of nude mice brains. Tumor growth is evaluated via bioluminescence (BLI) before being partially resected via fluorescent guidance 11 days after implant. A 25 μg-equivalent dose of Ace-ERL is then introduced in the resection cavity. On days 3, 5, 7, and 9 after resection, mice are given 5 mg/kg dose of TMZ interperitoneally (I.P.). Mice are monitored for tumor growth and survival. (B-E) BLI for mice following resection and (B) no treatment (No Tx, *n* = 6), (C) 25 μg Ace-ERL (*n* = 10), (D) 5 mg/kg I.P. TMZ (*n* = 7), or (E) combination treatment of Ace-ERL + TMZ (Combo, *n* = 14). (F) Kaplan-Meier survival curve where **p < 0.01, ***p < 0.001, and ****p < 0.0001 by log-rank (Mantel-Cox) test, reported in Supplemental Table S3. Median survival indicated in table under legend.

To assess the therapeutic efficacy of Ace-ERL added to the GBM standard of care, we adopted a clinically relevant murine model including GBM resection and recurrence for in vivo evaluation. [58] To do this, we first established a TMZ dosing regimen for mice bearing a PDx model (GBM8) which mirrors the modest survival benefit observed in standard of care treatment (Fig. 5A). The preliminary monotherapy studies identified that intraperitoneal (I.P.) administration of 5 mg/kg TMZ on days 3, 5, 7, and 9 post-GBM8 tumor resection extended median survival (MS) by approximately 30 %, approximating the ~2.5-month survival advantage reported in clinical settings (Supplemental Fig. S4A–B). [59]

For evaluating the combination of Ace-ERL and TMZ following GBM8 tumor resection, all mice received tumor resection surgery and either no treatment (No Tx), 25 μg of ERL from local administration of Ace-ERL scaffold, 5 mg/kg I.P. TMZ, or a combination of Ace-ERL + I.P. TMZ (Combo). Efficacy was assessed by monitoring tumor BLI signal and body weight/condition following resection (Fig. 5B–E, Supplemental Fig. S5). All mice receiving I.P. TMZ exhibited a reduction in BLI signal during the treatment period, with tumor recurrence typically observed after Day 14 (Fig. 5D–E). While significant BLI signal reduction was not evident in the Ace-ERL monotherapy group, mice treated with Ace-ERL demonstrated a significantly extended MS of 42 days, compared to 36 days in untreated controls (*p* < 0.01; Fig. 5C, F). Likewise, TMZ alone significantly prolonged survival by 12 days over controls (MS = 48 days; *p* < 0.001). Strikingly, the combination of Ace-ERL and I.P. TMZ yielded the most pronounced survival benefit, achieving an MS of 55 days—a 53 % improvement over untreated controls (*p* < 0.0001). This enhancement was significant relative to both monotherapies (*p* < 0.0001 vs. Ace-ERL; *p* < 0.001 vs. TMZ), confirming the therapeutic advantage of combining localized EGFR inhibition via Ace-ERL with I.P. TMZ administration in this clinically relevant GBM model. Log-rank statistics from the efficacy study are reported in Supplemental Table S3.

### 3.5. The in vivo benefit of Ace-ERL + TMZ demonstrates additive effects for improving progression free survival

**Fig. 6.**
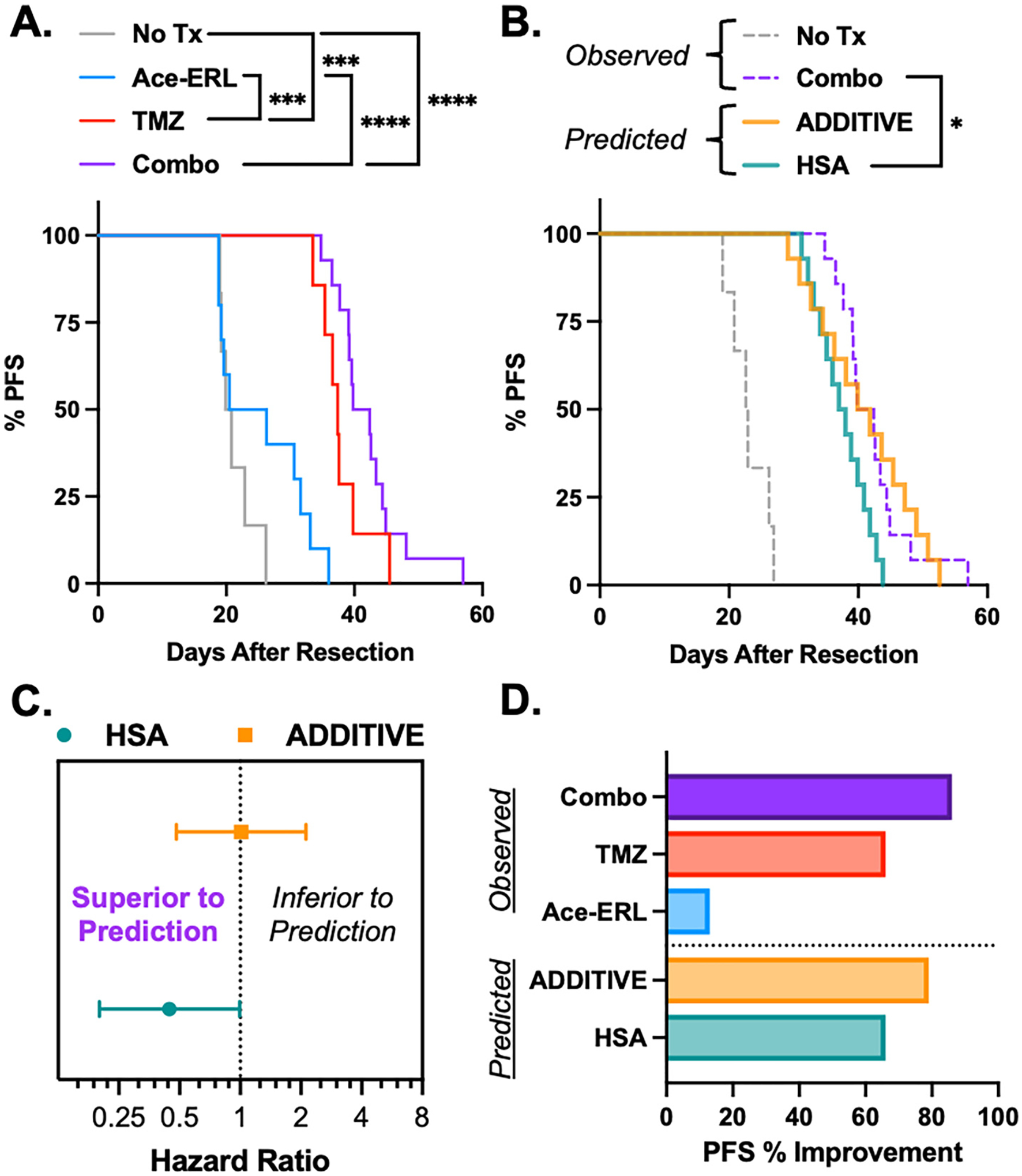
Combination effects of Ace-ERL with TMZ on progression free survival (PFS). (A) Kaplan-Meier curves for PFS from bioluminescence determined in efficacy study. PFS defined as a 200-fold increase in BLI. (B) Kaplan-Meier curves for the observed and predicted PFS using Additive and Highest-Single Agent (HSA) models. *p < 0.05, ***p < 0.001, ****p < 0.0001 by log-rank (Mantel-Cox) test. (C) Hazard ratio (HR) of the predicted models with respect to the observed PFS of combination (Combo) treatment where HR < 1 means the observed effects cannot be explained solely by the model and HR ≥ 1 means that the model is representative of the observed effects. Error bars are the 95 % confidence interval. (D) Observed and predicted % improvement in median PFS relative to untreated controls.

Although the combination of Ace-ERL and TMZ significantly improved overall survival compared to either monotherapy, we sought to further explore this benefit—specifically, whether the combination of Ace-ERL and TMZ synergized to reduce tumor recurrence in vivo. To address this, we evaluated progression-free survival (PFS), defined by a 200-fold increase in tumor bioluminescent signal over Day 1, in mice from the survival study (Fig. 6A). Interestingly, Ace-ERL monotherapy did not significantly improve PFS compared to untreated controls (*p* = 0.169), whereas TMZ alone resulted in a significant extension of PFS (p < 0.001). Moreover, TMZ significantly outperformed Ace-ERL in extending PFS (p < 0.001). While mice receiving the combination therapy showed improved PFS compared to both untreated (p < 0.0001) and Ace-ERL-treated mice (p < 0.0001), this improvement was not statistically significant when compared to TMZ alone (*p* = 0.057).

To better understand the underlying mechanisms contributing to this Combo therapeutic efficacy, we applied a framework developed by Hwangbo et al., who analyzed clinical PFS data from 37 FDA-approved drug combinations that had previously been described as ‘synergistic’ in preclinical studies. [60] Their approach used two benchmark models—Highest Single Agent (HSA) and Drug Additivity—to evaluate whether observed PFS benefits could be explained by the either of the two defined effects (Eqs. 2 and 3). Using this framework, we predicted the expected PFS outcomes for HSA and additive models based on our in vivo results and compared them to the observed PFS from the efficacy study (Fig. 6B). The observed PFS was significantly greater than the HSA model prediction (*n* = 14, *p* < 0.05), indicating that the combination effect exceeds what would be expected from the best-performing monotherapy (TMZ) alone. However, there was no significant difference between the observed and additive model predictions (*p* = 0.976), suggesting the combined benefit could be largely explained by additive effects.

This conclusion is further supported by hazard ratio (HR) analysis comparing observed versus predicted PFS (Fig. 6C). The HR for the HSA model was <1 (HR = 0.445), reinforcing that the combination performed better than the HSA expectation. In contrast, the HR for the additive model was approximately 1 (HR = 1.01), suggesting the observed effect closely matched the additive prediction. These trends become even more apparent when directly comparing median PFS improvements across observed treatments and the predictive models (Fig. 6D). In vivo, TMZ monotherapy extended median PFS by 66 %, while Ace-ERL alone provided a modest 13 % improvement over untreated controls. The HSA model, reflecting the best single-agent benefit, also predicted a 66 % increase. In contrast, the additive model predicted a 79 % improvement, combining the contributions of both agents. Notably, the observed combination therapy achieved an 86 % increase in median PFS—surpassing the additive model by 7 %. These findings suggest that although the additivity model closely captures the effects seen in vivo, there may be modest synergistic interactions contributing to the observed enhanced therapeutic effect from this combination treatment.

## 4. Conclusions

GBM remains one of the most deadly and aggressive brain tumors, with a critical need for new and more effective treatment strategies. Tumor heterogeneity, acquired drug resistance mechanisms, and physiological barriers such as the BBB all challenge the success of alternative treatments in outperforming the standard of care. [61] Therefore, clinical relevance and translational potential must be considered early in the preclinical pipeline. Moreover, supplementing the established standard of care presents a promising strategy to improve outcomes and address current challenges, but this approach requires thorough preclinical evaluation to determine whether a given drug combination offers a meaningful therapeutic benefit. While prior combination studies have shown preclinical promise, most have failed to reach clinical implementation, underscoring the need for innovative therapeutic solutions.

In this study, we sought to enhance the standard of care through localized delivery of a targeted therapy using electrospun acetalated dextran (Ace-DEX) scaffolds. This platform enables the incorporation of TMZ-synergizing agents—including those that do not efficiently cross the BBB—while also permitting localized, controlled drug release for optimum dosing. We validated differences in TMZ sensitivity across a panel of molecularly diverse cell lines which recapitulates clinical heterogeneity and highlights the benefit of patient-derived models over commonly used human models for preclinical assessment. Using the zero-interaction potency (ZIP) model, we identified erlotinib (ERL), an EGFR inhibitor, as the most synergistic partner with TMZ across the varied panel of GBM models. When formulated into Ace-DEX scaffolds (Ace-ERL) and locally administered into a resection cavity, ERL demonstrated sustained release for up to one week, maintaining therapeutic levels at the target site of tumor recurrence. This localized delivery, in combination with systemically administered TMZ, significantly extended survival in a clinically relevant, patient-derived mouse model, demonstrating a strategy to improve outcomes of combination therapy in GBM.

Although in vivo outcomes largely reflected additive effects, this result is consistent with clinical findings reported by Hwangbo et al., who noted that 95 % of FDA-approved oncology drug combinations result in additive or sub-additive interactions. [60] The observed ZIP synergy score of 5.90 premising in vivo evaluation reflects modest synergy, further supporting the translational relevance of our findings. Looking forward, future studies will explore higher-order combinations and integrate other mechanisms for GBM treatment to enhance therapeutic efficacy. [62] Further studies evaluating scaffolds with higher % CAC and thus longer release rates will also aim to uncover the implications of sustained delivery of targeted combination therapy. Collectively, this work highlights the promise of Ace-DEX scaffolds as a platform for delivering synergistic therapies and underscores the value of clinically grounded approaches in developing new treatment options for GBM.

## Supplementary Material

MMC1

Appendix A. Supplementary data

Supplementary data to this article can be found online at https://doi.org/10.1016/j.jconrel.2025.114225.

## Figures and Tables

**Table 1:** Electrospun Ace-ERL Formulations. Experimental ERL loading (% wt. ERL/scaffold) of electrospun scaffolds. Average fiber diameter measurements of *n* = 30 fibers from representative SEM images. Interpolated time (days) for 50 % ERL release from the linear regression trendlines in Fig. 4A–B. Data is presented as average ± standard deviation.

%CAC (Fig. 4 ID)	47 % CAC (A)	50 % CAC (B)
ERL % Load (wt.)	10.6 % ± 0.4 %	9.53 % ± 1.04 %
Fiber Diameter (μm)	1.03 ± 0.02	0.639 ± 0.124
Time_50_ (days)	4.99	30.3

## Data Availability

Data will be made available on request.

## Abbreviations:

GBM: glioblastoma *multiforme*
Ace-DEX: acetalated dextran
CAC: cyclic acetal coverage
TMZ: temozolomide
ERL: erlotinib
SFN: sorafenib
BUP: buparlisib
DBR: dabrafenib
TRM: trametinib
EVR: everolimus
IDSN: idasanutlin
RBC: ribociclib
MGMT: O^6^-methylguanine-DNA methyltransferase
RTK: receptor tyrosine kinase
EGFR: epidermal growth factor receptor
TCGA: the Cancer Genome Atlas
BCNU: carmustine
Ace-ERL: Erlotinib-loaded Acetalated Dextran electrospun scaffold
Combo: combination treatment
MS: median survival
PFS: progression free survival
PDx: patient-derived GBM xenograft
Hu: human GBM model
Mu: murine GBM model
BBB: blood-brain barrier
BLI: bioluminescent imaging
HSA: highest single agent
ZIP: zero-interaction potency
