# Supplementary material for: Biodegradable nanofibrous scaffolds enhance standard of care for glioblastoma via localized targeted therapy
Source: J Control Release. Author manuscript; Available in PMC 2025 Sep 25. (PMC12459348; doi:10.1016/j.jconrel.2025.114225)
Supplement: MMC1 [file NIHMS2111581-supplement-MMC1.docx]

**Supplemental: Biodegradable Nanofibrous Scaffolds Enhance Standard of Care for Glioblastoma via Localized Targeted Therapy**

Ryan N. Woodring^1^, Elizabeth G. Graham-Gurysh^1^, Sophie E. Mendell^1^, Kevin E. Shilling^1^, Nicole Rose Lukesh^1^, Katie A. Hipp^4^, William C. Zamboni^4^, Eric M. Bachelder^1^, Kristy M. Ainslie^1,2,3,*^

^1^Division of Pharmacoengineering & Molecular Pharmaceutics, Eshelman School of Pharmacy, UNC, Chapel Hill, NC, USA

^2^Department of Biomedical Engineering, NC State/UNC, Chapel Hill, NC, USA

^3^Department of Microbiology and Immunology, School of Medicine, UNC, Chapel Hill, NC, USA

^4^Division of Pharmacotherapy and Experimental Therapeutics, Eshelman School of Pharmacy, UNC, Chapel Hill, NC, USA

*Corresponding author:

Kristy M. Ainslie

Fred Eshelman Distinguished Professor

Chair, Division of Pharmacoengineering & Molecular Pharmaceutics

UNC Eshelman School of Pharmacy

4012 Marsico Hall, 125 Mason Farm Road

Chapel Hill, NC 27599, United States

[ainsliek@email.unc.edu](mailto:ainsliek@email.unc.edu)

**Supplemental Contents:**

- **Supplemental Equation S1:** ZIP Synergy Quantification
- **Supplemental Equation S2:** Summary ZIP Synergy Score
- **Supplemental Equation S3:** Scaffold Drug-loading (% wt.)
- **Supplemental Equation S4:** Scaffold (%) Mass Loss
- **Supplemental Tables:** References for Cell Line Characterization
  - **Table S1:** MGMT Promoter Methylation Status
  - **Table S2:** EGFR Status
  - **Table S3:** Log-Rank Statistical Analysis of Survival Study
- **Supplemental Figure S1:** Ace-DEX Synthesis and Characterization
- **Supplemental Figure S2:** In Vitro Viability Matrices
- **Supplemental Figure S3:** In Vivo Drug Release and Tolerability
- **Supplemental Figure S4:** TMZ Standard of Care Pilot Studies
- **Supplemental Figure S5:** Representative BLI and Mouse Weights from Efficacy Study
- **Supplemental References**

**Supplemental Equation S1**

ZIP Synergy, $\boldsymbol{\delta}=\frac{1}{2}\left[ \frac{\frac{1}{1+\left( \frac{m_{B}}{X_{B}} \right)^{\lambda_{B}}}+\left( \frac{X_{A}}{m_{A\leftarrow B}} \right)^{\lambda_{A\leftarrow B}}}{1+\left( \frac{X_{A}}{m_{A\leftarrow B}} \right)^{\lambda_{A\leftarrow B}}}+\frac{\frac{1}{1+\left( \frac{m_{A}}{X_{A}} \right)^{\lambda_{A}}}+\left( \frac{X_{B}}{m_{B\leftarrow A}} \right)^{\lambda_{B\leftarrow A}}}{1+\left( \frac{X_{B}}{m_{B\leftarrow A}} \right)^{\lambda_{B\leftarrow A}}} \right]-\left[ \frac{\left( \frac{X_{A}}{m_{A}} \right)^{\lambda_{A}}}{1+\left( \frac{X_{A}}{m_{A}} \right)^{\lambda_{A}}}+\frac{\left( \frac{X_{B}}{m_{B}} \right)^{\lambda_{B}}}{1+\left( \frac{X_{B}}{m_{B}} \right)^{\lambda_{B}}}-\frac{\left( \frac{X_{A}}{m_{A}} \right)^{\lambda_{A}}}{1+\left( \frac{X_{A}}{m_{A}} \right)^{\lambda_{A}}}\cdot\frac{\left( \frac{X_{B}}{m_{B}} \right)^{\lambda_{B}}}{1+\left( \frac{X_{B}}{m_{B}} \right)^{\lambda_{B}}} \right]$

Zero-interaction potency (ZIP, δ) Synergy quantification between drug A and drug B using parameters from the 4-parameter log-mean trendlines where x_A/B_ is a given concentration of drug A/B; m_A/B_ is the concentration of drug A/B achieving 50% viability (or 50% inhibitory concentration, IC_50_); λ_A/B_ is the sigmoidicity slope parameter; m_A/B←B/A_ is the IC_50_ of x_A/B_ added to drug B/A; and λ_A/B←B/A_ is the sigmoidicity of x_A/B_ added to drug B/A.

**Supplemental Equation S2**

Summary ZIP Synergy, $\boldsymbol{\Delta}= \frac{1}{n} \sum_{i=1}^{n} \delta_{\iota}$

Where n is the number of combinations in a 2-drug increasing-dose matrix and δ_i_ is the ZIP synergy score for the i^th^ combination.

**Supplemental Equation S3**

$Scaffold Drug \% Loading= \frac{Concentration of drug (\frac{mg}{mL})}{Concentration of scaffold (\frac{mg}{mL})}\times100\%$

Ace-DEX scaffold dissolved at 1 mg/mL of DMSO and concentration of drug determined via absorbance at 330nm, compared to a standard curve.

**Supplemental Equation S4**

$Scaffold \% mass retained= \frac{Mass of scaffold at time (t)}{Initial Mass of scaffold (t=0)}\times100\%$

Scaffold mass retained calculated from in vitro drug release study where pre-weighed scaffold samples were incubated for time, t, before being washed in basic water, lyophilized, and reweighed to determine mass of scaffold remaining.

**Supplemental Tables**

**Supplemental Table S1: MGMT promoter methylation status reported in literature.** Available reports in literature of the methylation status of O^6^-methylguanine-methyltransferase (MGMT) promoter in the panel of GBM cell lines. Method of methylated or unmethylated promoter detection sourced from respective references. MS-PCR = methylation-specific polymerase chain reaction.[1-6]

| Model | Cell Line | MGMT Promoter Status | Method of Detection | Ref. |
| --- | --- | --- | --- | --- |
| Patient Derived (PDx) | **GBM8** | Methylated | MS-PCR | 1 |
| Human (Hu) | **U87** | Methylated | Western Blot | 2 |
|  | **U251** | Methylated | Pyrosequencing (MSP) | 3 |
|  | **LN18** | Unmethylated | Western Blot | 4 |
|  | **LN229** | Methylated | Western Blot | 5 |
| Murine (Mu) | **GL261** | Unmethylated | Western Blot | 6 |

**Supplemental Table S2: EGFR status of cell lines reported in literature.** Available reports in literature of the status of epidermal growth factor receptor (EGFR) in the panel of GBM cell lines. Status includes amplification or wild type (unmodified). RT-PCR = reverse transcriptase polymerase chain reaction, SDS-PAGE = Sodium Dodecyl Sulfate Polyacrylamide Gel Electrophoresis.[7-12]

| Model | Cell Line | EGFR Status | Method of Detection | Ref. |
| --- | --- | --- | --- | --- |
| Patient Derived (PDx) | **GBM8** | Amplified | Immunoblotting | 7 |
|  | **U3013PN** | Amplified | DNA copy number alteration | 8 |
|  | **U3017CL** | Amplified | DNA copy number alteration | 8 |
|  | **U3031MS** | Amplified | DNA copy number alteration | 8 |
| Human (Hu) | **U87** | Wild Type | RT-PCR | 9 |
|  | **U251** | Amplified | SDS-PAGE | 10 |
|  | **LN18** | Wild Type | RNAseq/Western Blot | 11 |
|  | **LN229** | Wild Type | RNAseq/Western Blot | 11 |
| Murine (Mu) | **CT2A** | Wild Type | Western Blot | 12 |
|  | **GL261** | Wild Type | Western Blot | 12 |

**Supplemental Table S3: Summary of Log-Rank (Mantel-Cox) statistical analysis from efficacy study.** Table includes pairwise comparison between treatment groups, cohort sizes, median survival times, log-rank statistic (p-value), hazard ratio, and associated 95% confidence interval (CI). Results are appended in **Figure 5F**.

| Comparison | Group sizes (n) | Median Survival (days) | Log-rank p-value | Hazard Ratio | 95% CI |
| --- | --- | --- | --- | --- | --- |
| Ace-ERL vs. No Tx | 10 vs. 6 | 42 vs. 36 | 0.0036 (**) | 0.2960 | 0.07526 – 1.164 |
| TMZ vs. No Tx | 7 vs. 6 | 48 vs. 36 | 0.0003 (***) | 0.2240 | 0.05106 – 0.9829 |
| Combo vs. No Tx | 14 vs. 6 | 55 vs. 36 | <0.0001 (****) | 0.1617 | 0.02728 – 0.9591 |
| TMZ vs. Ace-ERL | 7 vs. 10 | 48 vs. 42 | 0.1974 (ns) | 0.5715 | 0.2198 – 1.486 |
| Combo vs. Ace-ERL | 14 vs. 10 | 55 vs. 42 | <0.0001 (****) | 0.2198 | 0.06835 – 0.7070 |
| Combo vs. TMZ | 14 vs. 7 | 55 vs. 48 | 0.0003 (***) | 0.2385 | 0.05962 – 0.9539 |

**Supplemental Figures**


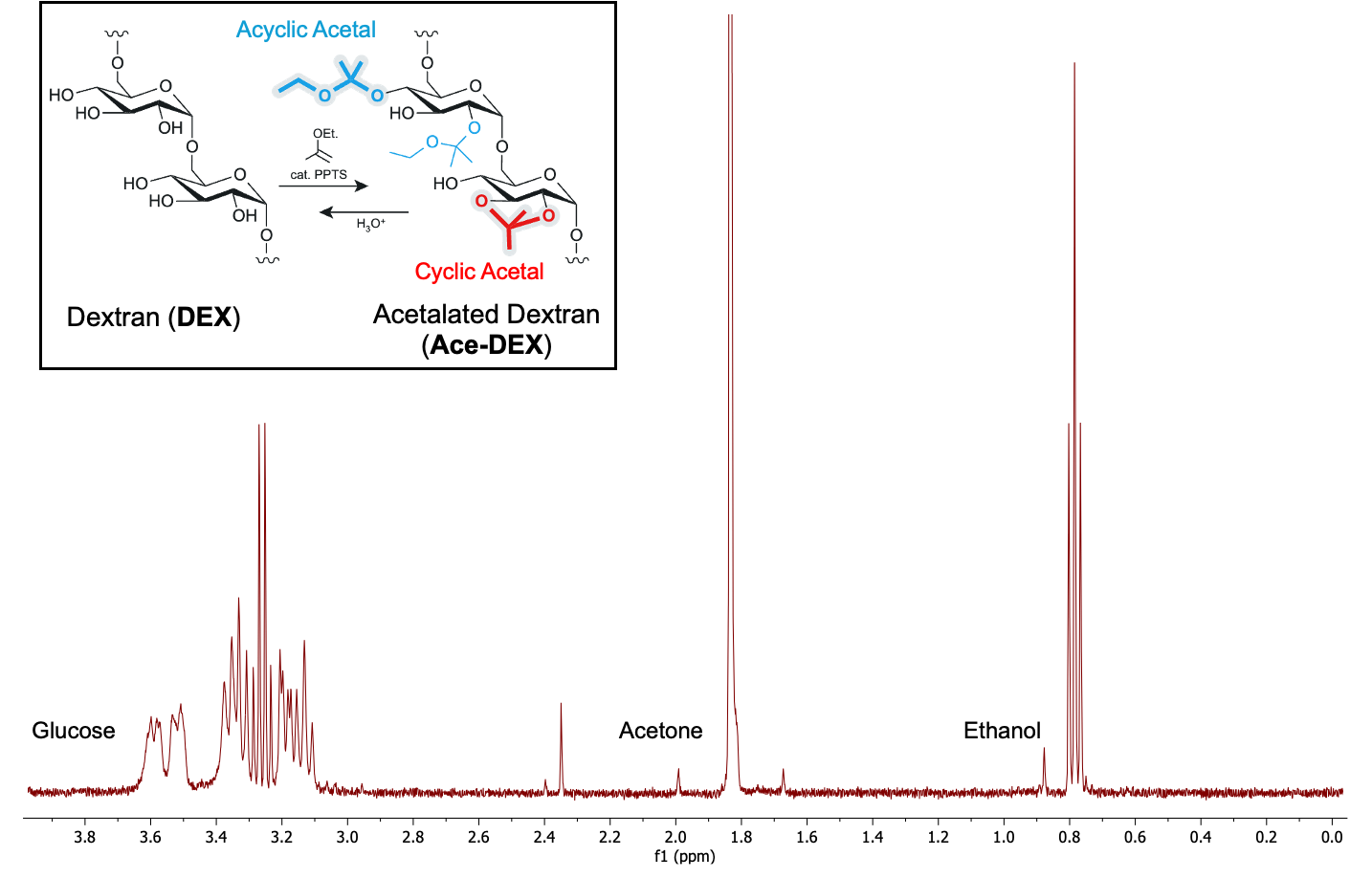


**Supplemental Figure S1: Example ^1^H-NMR Spectrum for Synthesized Ace-DEX.** Polymer was synthesized following the reaction scheme shown in the inlet image. The reaction of dextran (DEX) with 2-ethoxypropene and the acid catalyst pyridinium *p*-toluenesulfonate (PPTS) was executed for 20 minutes. The resulting polymer was purified and dissolved at 2.5 mg/mL in a solution of deuterium oxide with 10% (vol./vol.) deuterium chloride (Sigma). Integrated peaks of degradation byproducts, acetone and ethanol, were normalized to glucose which were used to calculate a 50% relative cyclic-to-acyclic acetal coverage (%CAC).


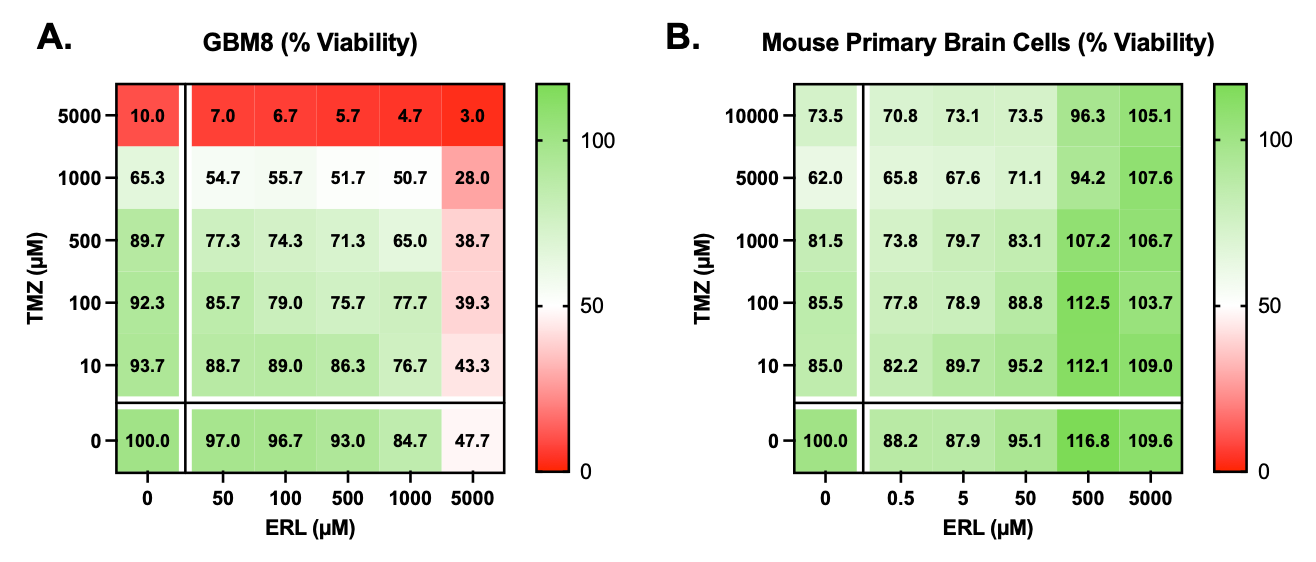
**Supplemental Figure S2: In vitro viability matrices of ERL + TMZ.** Heat maps showing percent of viable cells following treatment of ERL + TMZ combination matrix in cultured **(A)** GBM8 cells and **(B)** cells from primary brain tissue of healthy C57BL/6 mice.

**Supplemental Figure S3: In vivo release of 100 µg ERL from localized Ace-ERL fast scaffold. (A)** Release kinetics of 1mg Ace-ERL scaffolds (~100 µg ERL) assessed in vitro following the in vivo pharmacokinetic studies. Nude female mice received 100µg of ERL from (1mg) of 47% CAC Ace-ERL scaffolds in a mock tumor resection cavity (n=3 per time point). At each time point, remaining scaffolds were physically removed from the resection cavity, washed in basic water, lyophilized, and assessed for drug retained as described in the methods. **(B)** Averaged normalize weights of mice in the days following resection and scaffold treatment (Normalized to mass pre-surgery). All data are mean ± standard deviation.


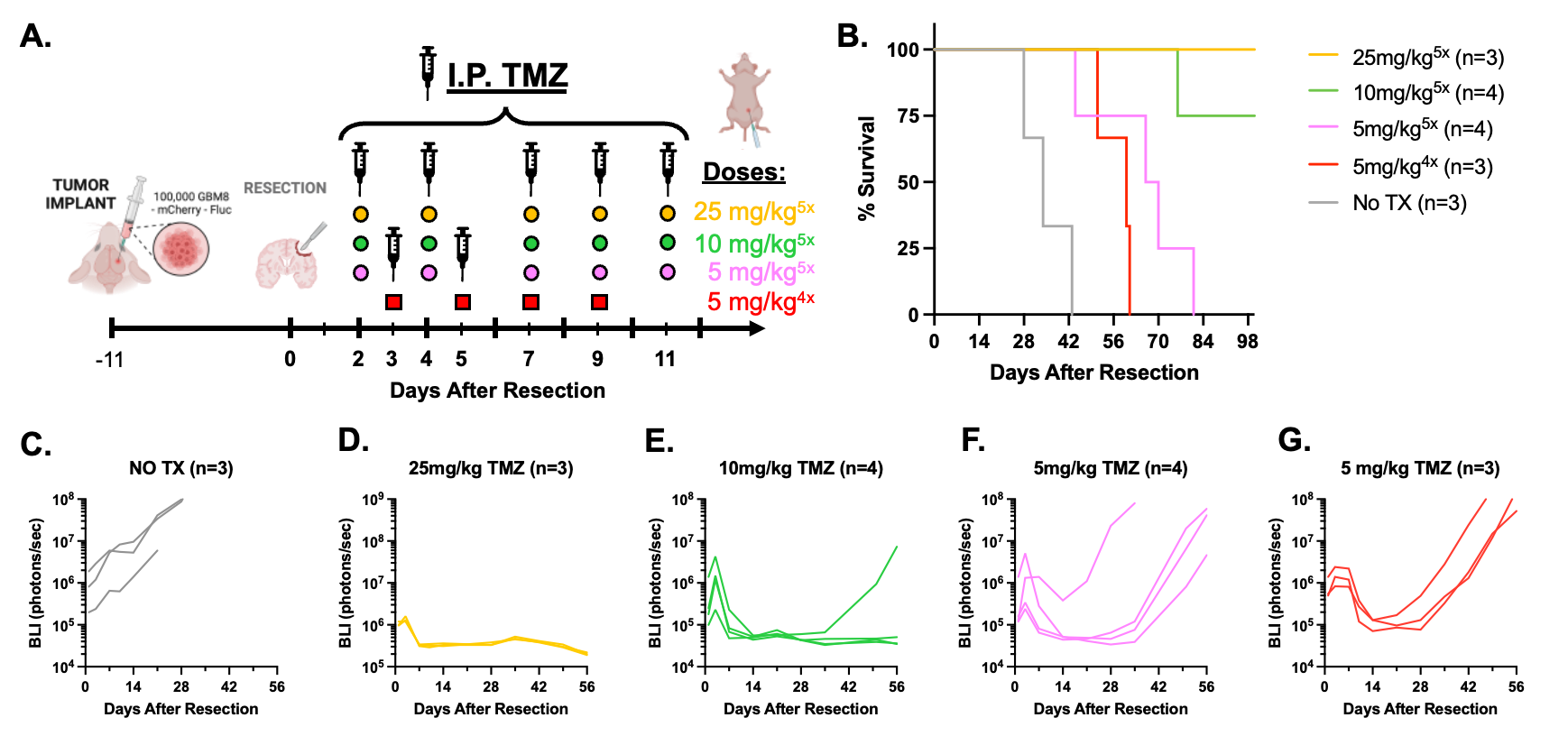


**Supplemental Figure S4: In vivo temozolomide (TMZ) dose optimization. (A)** Schematic of in vivo GBM8 tumor mouse model of resection and recurrence with systemic TMZ. GBM8-mCherry-Fluc cells are first implanted orthotopically 11 days before resection. Following resection, cohorts of mice received either no treatment (No Tx), or 5 (‘5x’, circles) or 4 (‘4x’, squares) doses of intraperitoneal (I.P.) TMZ (indicated by color). 5x doses included 25mg/kg (yellow), 10mg/kg (green), 5mg/kg (pink), and 4x doses included 5mg/kg (red) on the respective days following resection. **(B)** Kaplan-Meier survival curve for the dose optimization study. **(C-G)** Bioluminescent imaging (BLI) signal of tumors following resection for the respective treatments.

**Supplemental Figure S5. Representative Tumor Bioluminescence (BLI) and Treatment Tolerability. (A)** Tumor BLI (photons/sec) images shown for representative groups of mice from each treatment group including no treatment (No TX, n=6), 25 µg dose of erlotinib (ERL) from a localized 47% CAC Ace-DEX scaffold (Ace-ERL, n=10), 4 doses of 5mg/kg I.P. temozolomide (TMZ, n=7), and combination treatment of Ace-ERL and I.P. TMZ (Combo, n=14). Indicated images are listed respective to the day after resection of which BLI was assessed. ‘-‘ denotes mouse euthanized prior to imaging. **(B)** Mean ± standard error of the mean of mice weights normalized to Day 0 weight for each treatment group.

**Supplemental References**

[1] G.J. Kitange, B.L. Carlson, A.C. Mladek, P.A. Decker, M.A. Schroeder, W. Wu, P.T. Grogan, C. Giannini, K.V. Ballman, J.C. Buckner, C.D. James, J.N. Sarkaria, Evaluation of MGMT promoter methylation status and correlation with temozolomide response in orthotopic glioblastoma xenograft model, J Neurooncol, 92 (2009) 23-31.

[2] G.Z. Yi, G. Huang, M. Guo, X. Zhang, H. Wang, S. Deng, Y. Li, W. Xiang, Z. Chen, J. Pan, Z. Li, L. Yu, B. Lei, Y. Liu, S. Qi, Acquired temozolomide resistance in MGMT-deficient glioblastoma cells is associated with regulation of DNA repair by DHC2, Brain, 142 (2019) 2352-2366.

[3] M. Anan, R.F. Del Maestro, N. Hata, M. Fujiki, O(6) -methylguanine methyltransferase promoter methylation status of glioblastoma cell line clonal population, Neuropathology, 44 (2024) 41-46.

[4] T. Viel, P. Monfared, S. Schelhaas, I.B. Fricke, M.T. Kuhlmann, C. Fraefel, A.H. Jacobs, Optimizing glioblastoma temozolomide chemotherapy employing lentiviral-based anti-MGMT shRNA technology, Mol Ther, 21 (2013) 570-579.

[5] S. Zhu, J. Guo, L. Yu, J. Liu, J. Chen, J. Xin, Y. Zhang, J. Luo, C. Duan, Synergistic effect of cryptotanshinone and temozolomide treatment against human glioblastoma cells, Scientific Reports, 13 (2023) 21835.

[6] E.J. Ahn, Y.J. Kim, M.R. Akanda, S.J. Oh, T.Y. Jung, S. Jung, J.H. Lee, S.S. Kim, Y.Y. Jeong, H.H. Ha, H. Hyun, H. Kim, J.H. Rhee, K.K. Kim, K.H. Lee, K.S. Moon, Metastasis-enhancing protein KITENIN confers temozolomide resistance on glioblastoma with unmethylated MGMT via upregulation of cancer stem cell makers, Clin Transl Med, 14 (2024) e1804.

[7] H. Johnson, A.M. Del Rosario, B.D. Bryson, M.A. Schroeder, J.N. Sarkaria, F.M. White, Molecular characterization of EGFR and EGFRvIII signaling networks in human glioblastoma tumor xenografts, Mol Cell Proteomics, 11 (2012) 1724-1740.

[8] Y. Xie, T. Bergström, Y. Jiang, P. Johansson, V.D. Marinescu, N. Lindberg, A. Segerman, G. Wicher, M. Niklasson, S. Baskaran, S. Sreedharan, I. Everlien, M. Kastemar, A. Hermansson, L. Elfineh, S. Libard, E.C. Holland, G. Hesselager, I. Alafuzoff, B. Westermark, S. Nelander, K. Forsberg-Nilsson, L. Uhrbom, The Human Glioblastoma Cell Culture Resource: Validated Cell Models Representing All Molecular Subtypes, EBioMedicine, 2 (2015) 1351-1363.

[9] K. Mishima, T.G. Johns, R.B. Luwor, A.M. Scott, E. Stockert, A.A. Jungbluth, X.D. Ji, P. Suvarna, J.R. Voland, L.J. Old, H.J. Huang, W.K. Cavenee, Growth suppression of intracranial xenografted glioblastomas overexpressing mutant epidermal growth factor receptors by systemic administration of monoclonal antibody (mAb) 806, a novel monoclonal antibody directed to the receptor, Cancer Res, 61 (2001) 5349-5354.

[10] B. Stea, R. Falsey, K. Kislin, J. Patel, H. Glanzberg, S. Carey, A.A. Ambrad, E.J. Meuillet, J.D. Martinez, Time and dose-dependent radiosensitization of the glioblastoma multiforme U251 cells by the EGF receptor tyrosine kinase inhibitor ZD1839 (‘Iressa’), Cancer Letters, 202 (2003) 43-51.

[11] V. Patil, J. Pal, K. Somasundaram, Elucidating the cancer-specific genetic alteration spectrum of glioblastoma derived cell lines from whole exome and RNA sequencing, Oncotarget, 6 (2015).

[12] J.B. Iorgulescu, N. Ruthen, R. Ahn, E. Panagioti, P.C. Gokhale, M. Neagu, M.C. Speranza, B.K. Eschle, K.M. Soroko, R. Piranlioglu, M. Datta, S. Krishnan, K.B. Yates, G.J. Baker, R.K. Jain, M.L. Suvà, D. Neuberg, F.M. White, E.A. Chiocca, G.J. Freeman, A.H. Sharpe, C.J. Wu, D.A. Reardon, Antigen presentation deficiency, mesenchymal differentiation, and resistance to immunotherapy in the murine syngeneic CT2A tumor model, Frontiers in Immunology, Volume 14 - 2023 (2023).
